# Homomultimeric FAP Inhibitor-Based Radioligands for Cancer Theranostics: Design Principles, Structure–Function Relationships, and Preclinical Performance

**DOI:** 10.3390/molecules31122124

**Published:** 2026-06-16

**Authors:** Zhiyang Wu, Eleni Gourni, Sanjana Ballal, Pieter Van der Veken, Frank Roesch

**Affiliations:** 1 SCV Spezial-Chemikalien-Vertrieb GmbH, 10243 Berlin, Germany; 2 Department of Nuclear Medicine, Inselspital, Bern University Hospital, CH-3010 Bern, Switzerland; eleni.gourni@insel.ch; 3 Department of Nuclear Medicine, All India Institute of Medical Sciences, New Delhi 110029, India; mail.sanjanaballal87@gmail.com; 4 Laboratory of Medicinal Chemistry, Department of Pharmaceutical Sciences, University of Antwerp, 2610 Wilrijk, Belgium; pieter.vanderveken@uantwerpen.be; 5 Department of Chemistry–TRIGA Site, Johannes Gutenberg-University Mainz, 55128 Mainz, Germany

**Keywords:** homomultimeric, FAPI ligands, theranostics, fibroblast activation protein (FAP), radioligand therapy (RLT), dimerization, multimerization

## Abstract

Fibroblast activation protein (FAP) has emerged as a promising target for the development of cancer radiotheranostics due to its selective overexpression in cancer-associated fibroblasts (CAFs) within the tumor stroma. Affinity and selectivity refer to the binding affinities of FAP inhibitors toward FAP and related family members, whereas the accumulation of radiolabeled-FAP inhibitors varies by tumor type. Although monomeric FAP inhibitors (FAPIs) have shown extraordinary utility in diagnostic imaging, their clinical application in radiotherapy has been limited by short tumor retention times and heterogeneous uptake. To address these challenges, homomultimeric FAPI ligands—featuring two or more identical FAP-targeting motifs—have been developed with the aim of enhancing binding avidity and prolonging tumor residence. This review comprehensively examines the evolution of homomultimeric FAPI ligands, from molecular design and preclinical validation to early clinical implementation. We highlight how dimeric and higher-order multimeric constructs improve tumor retention and therapeutic efficacy compared to monomers, while also discussing the impact of linker chemistry, valency, and scaffold architecture on pharmacokinetics and targeting efficiency. Preclinical studies demonstrate that optimized dimers and trimers achieve superior tumor-to-background ratios and sustained tumor uptake, whereas excessive multimerization can lead to steric hindrance and reduced efficacy. Clinical data from pioneering studies using agents such as [^177^Lu]Lu-DOTAGA.(SA.FAPi)_2_ and [^177^Lu]Lu-DOTAGA.Glu.(FAPi)_2_ confirm prolonged tumor retention, encouraging therapeutic responses and a favorable safety profile in advanced cancers. However, translational challenges remain, including the need for better preclinical models that reflect stromal FAP heterogeneity, optimized radiometal–chelator pairs, and standardized dosing protocols for comparative clinical trials. Overall, homomultimeric FAPI ligands represent a significant advance in FAP-targeted theranostics, offering a robust platform for personalized cancer management.

## 1. Introduction

Fibroblast activation protein (FAP), a type II transmembrane serine protease, has received significant attention as a biomarker in oncology due to its selective overexpression in cancer-associated fibroblasts (CAFs) within the tumor microenvironment (TME) of over 90% of epithelial cancers [1,2,3]. FAP plays a critical role in extracellular matrix (ECM) remodeling [4], facilitating tumor invasion [5], metastasis [6], and immunosuppression through its dipeptidyl peptidase and endopeptidase activities [7]. Its relatively low expression in normal adult tissues—except during wound healing [8] or fibrosis [9]—establishes FAP as an ideal target for tumor-selective radiopharmaceuticals [10]. Following the identification of the highly selective FAP inhibitor UAMC-1110 [11], the first FAP inhibitor (FAPI) ligands, including FAPI-04, FAPI-46, and FAPI-74, were subsequently developed [2]. These radiolabeled derivatives incorporate DOTA-based chelators for radiometal complexation and have been optimized for PET imaging (^68^Ga or ^18^F) or radiotherapy (^177^Lu, ^90^Y, and ^225^Ac), with variable pharmacokinetic profiles. These initial agents, each comprising a single targeting vector (i.e., monomeric radioligands), demonstrated rapid tumor uptake, low background signal, and promising efficacy in clinical PET imaging [12,13,14]. However, their clinical application for therapy was limited by transient tumor retention (often <24 h) and heterogeneous uptake in lesions exhibiting variable stromal density [13,15,16]. These limitations prompted the development of advanced chemical designs for FAP-targeted radiotherapy.

**Figure 1:**
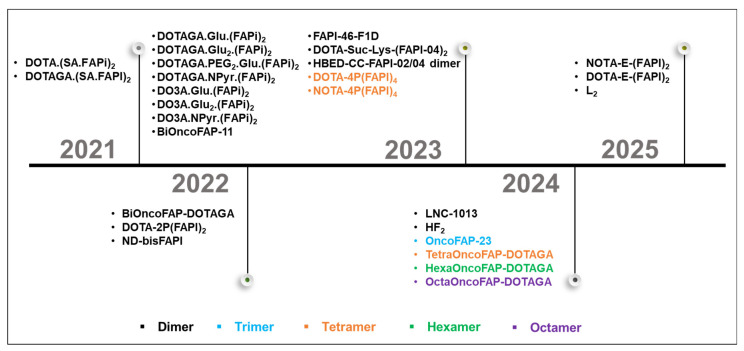
Publication timeline of homomultimeric FAPI radioligands. Derivatives beyond dimers are indicated by colors. HF_2_: (HYNIC-BiOncoFAP), L_2_: (PEG_4_.(FAPI-46)_2_).

An interesting strategy to prolong tumor retention involves the development of multimeric radioligands, a concept previously validated in 2019 by a comparative study of mono- and bivalent PSMA-targeting PET tracers [17]. In 2020, Moon et al. developed the first homodimeric FAPI ligands, engineered by conjugating two identical FAPI moieties via squaramide linkers, representing a paradigm shift in targeting FAP-expressing stroma. The dimeric design significantly increased tumor accumulation without compromising affinity or selectivity for FAP in comparison with the monomeric derivative [18]. The first-in-human application of this ^177^Lu-labeled dimeric construct [^177^Lu]Lu-DOTAGA.(SA.FAPi)_2_ for patient treatment was reported at the 2021 SNMMI Annual Meeting in Washington, D.C. The results have confirmed its potential to expand the theranostic utility of the FAPI-based radiotherapeutics, especially in malignancies characterized by dense stromal components [19]. Other homodimeric and homotrimeric FAPI variants have developed shortly after, in pre-clinical studies confirming the concept of FAPI homomultimerization in terms of improvements in tumor residence times, tumor-to-background ratios and radiation dosimetry [20,21]. This progress has catalyzed the development of even higher-order multimers such as tetramer, hexamer, and octamer to achieve further improvements in tumor uptake and retention [22,23]. Meanwhile, a number of those derivatives have been published, and Figure 1 illustrates the history of FAPI-homomultimers.

This review focuses on the strategy of FAPI multimerization, specifically on FAPI homodimers, and comprehensively examines the evolution of homomultimeric FAPI ligands from molecular design to clinical implementation. It begins by defining key terms essential for understanding tumor retention and then systematically presents all relevant data—starting with chemical design, followed by in vitro cellular evaluation, in vivo and ex vivo assessment in animal models, and finally clinical data. We discuss (1) structural innovations that enhance FAP affinity, selectivity, and tumor residence time; (2) preclinical evidence supporting diagnostic and therapeutic applicability; (3) clinical data demonstrating utility across various solid tumors; and (4) unresolved challenges regarding animal models, toxicity, and clinical scalability. The review concludes with a critical discussion and an optimistic outlook, aiming to highlight opportunities for personalizing cancer management through FAP-directed multimodality approaches within the broader landscape of stroma-targeted theranostics.

### Tumor Residence Time: Definitions

The intention to improve therapeutic efficacy compared to the respective monomeric forms may appear obvious to the reader, and indeed, this principle has been recognized for over a century. In their paper entitled “Multi-scale modeling of drug binding kinetics to predict drug efficacy,” Clarelli et al. [24] refer to Paul Ehrlich, who already noted in 1913 [25] that “a drug is efficacious only so long as it binds to, and modulates the action of, its physiological target.”

In fact, medicinal chemistry has a wide range of options for optimizing a ligand’s therapeutic efficacy in systematic preclinical studies—both in terms of PK and PD. However, traditional approaches typically focus on structure-affinity correlations, with an emphasis on the binding equilibrium between a drug molecule (referred to hereunder as L) and its biological target T (in the present context synonym to T = tumor). The central parameter has traditionally been the binding or equilibrium constant K_d_.

In many cases of 1:1 binding of a drug molecule L to its respective biological target, a certain, sometimes relatively short residence time of the ligand at the target is observed, despite the ligand’s high-affinity binding (K_d_ value) to the target. It is surprising that the “target residence time” of the ligand has only received greater attention in recent years.

It is therefore worthwhile to review the key concepts of pharmacodynamic (PD) and pharmacokinetic (PK) models related to a drug molecule’s target residence time. It is advisable to use RA Copeland’s excellent 2016 paper “The drug-target residence time model: a 10-year retrospective” [26], which focuses on small drug molecules, as a guide. Mathematical analyses of enzyme inhibition kinetics (binding vs. dissociation) can be found in Morrison and Walsh [27].

The interaction between the drug molecule L and its target T is implicitly considered as a ligand-target EQUILIBRIUM:

(1) $$\mathrm{Ligand}\left(L\right)+\mathrm{Target}\left(T\right)\overset{k_{\mathrm{on}}}{\underset{k_{\mathrm{off}}}{⇌}}\mathrm{Complex}\left(\mathrm{LT}\right)$$

For this ligand-target complex, the EQUILIBRIUM CONSTANT K_d_, also defined as binding affinity, can be quantified as K_d_ (unit M, mol per liter). It is numerically equal to the ratio of the association rate k_on_ (units M^−1^ s^−1^) and the dissociation rate k_off_ (units s^−1^):

K_d_ = k_off_/k_on_. (2)

Measurements are performed in vitro, in a static setup or closed system; kinetic phenomena are typically not considered. (Terms derived from K_d_ include the half-maximal inhibitor concentration IC_50_, the effector concentration for half-maximal response EC_50_, and the inhibition constant K_i_).

The ASSOCIATION RATE k_on_ (units M^−1^ s^−1^) is generally regarded as a central parameter of the drug–molecule–target interaction and implicitly represents a fast and strong binding L + T → LT.

The DISSOCIATION RATE k_off_ (units s^−1^) defines the rate at which the bond between the two partners breaks; in the case of LT → L + T, i.e., reversible binding, this is an intrinsic characteristic of “equilibria.” The HALFLIFE of the ligand-target complex is given as t½ = ln2/k_off_.

The DRUG-TARGET RESIDENCE TIME τ is defined via the DISSOCIATION RATE k_off_, specifically as its reciprocal value: τ = 1/k_off_. It thus has the logical unit of time (s). While the equilibrium constant has traditionally been regarded as the key determinant of a drug’s efficacy, and in medicinal chemistry, the optimization of a lead compound is guided by the K_d_ value, the DRUG-TARGET RESIDENCE TIME (τ) has only recently begun to receive specific attention; see Copeland et al. [26] and Vauquelin et al. [28]. It does indeed seem obvious that under dynamic, in vivo conditions, both k_on_ and k_off_ must be examined more closely, essentially from the perspective of a “real-life scenario.” At the same time, it seems obvious that actual POTENCY—i.e., the therapeutic effect—is significantly influenced by the rate of dissociation from the target. The key message can be summarized as follows: “τ is more important than k_on_.” In other words, it is increasingly important to understand which types of L-T bonds slow down the dissociation of the complex.

Those phenomena naturally also apply to radiopharmaceutical chemistry and nuclear medicine. However, unlike the examples of PSMA, SSTR, and CXCR4 radiotherapeutics—where tumor residence time was not a limiting factor and did not guide development—FAP inhibitors present a unique challenge. First-generation FAP inhibitors exhibit short tumor retention on the order of hours, making them excellent diagnostic agents when labeled with ^68^Ga or ^18^F, but suboptimal for therapy when simply relabeled with therapeutic radionuclides such as ^90^Y, ^177^Lu, or ^225^Ac. These derivatives, therefore, perfectly reflected the classical understanding of radiotheranostics: in a given chelator-conjugated oncological vector (ligand), the radiometal is simply varied to switch from a diagnostic agent to an analogous therapeutic agent. Binding affinities, lipophilicity, and other relevant properties (including the residence time) remain unchanged.

However, this traditional understanding of radiotheranostics is being challenged by the emergence of FAP inhibitors as potential pan-tumor radio-theranostics: first-generation FAP inhibitors exhibit short tumor retention on the order of hours. In their ^68^Ga- or ^18^F-labeled variants, they are perfect diagnostic agents and allow for PET studies as early as 1 h after injection. The simple substitution of the trivalent radiometal ^68^Ga with ^90^Y, ^177^Lu, and ^225^Ac, etc., however, proves to be suboptimal for therapeutic applications. For FAP inhibitors labeled with therapeutic radioisotopes having half-lives of several days, suboptimal doses are delivered to the tumor.

Nevertheless, the nuclear medicine community has high hopes for the availability of therapeutic FAPI derivatives. Radiopharmaceutical chemistry is actively engaged in various methodological approaches aimed at prolonging the half-life of therapeutic FAPI derivatives. These include conventional coupling with FAPI-nonspecific albumin-binding units, which prolong the retention time in blood plasma, conjugation with FAPI-nonspecific covalently binding structures, and optimization of the FAPI itself. Historically, however, the strategy of multimerization of FAPI to prolong tumor retention time was the initial approach to systematically extend the half-life of a FAPI lead compound. The initial concept was that doubling (homodimers) or tripling (homotrimers), etc., would exponentially prolong the retention time of the homomultimers compared to the (too) short retention time of the respective FAPI monomer. The working hypothesis was that, due to the intensification and simultaneity of the various chemical bonds and interactions between individual components of the FAPI monomers on the one hand and individual components of the 3-dimensional amino acid sequence of the target protein FAP on the other, synergistic effects would delay the dissociation of the binding equilibrium between the FAP inhibitor (ligand L) and FAP (target T).

It is worth noting that all homomultimeric FAP inhibitor radioligands published to date have been developed by academic radiochemistry groups rather than by pharmaceutical companies. This may explain the observed heterogeneity in nomenclature, inconsistent linker labeling (e.g., L_1_, L_2_, and HF_2_), and the limited availability of standardized clinical data across studies.

## 2. Molecular Design

A FAP targeting ligand is typically composed of three core structural elements: chelator, linker/spacer, and molecular vector, such as an antibody, peptide, or FAP inhibitor (FAPI) [29,30,31]. The chelator firmly coordinates the radiometals, with the principal design of (1,4,7,10-tetraazacyclododecane-1,4,7,10-tetrayl) tetraacetic acid macrocycles (DOTA and its derivatives) being the most common choice of chelators [32]. The linker/spacer connects the chelator to the molecular vector and is critical for modulating the radioligand’s hydrophilicity and pharmacokinetics, while also influencing the spatial geometry of the radionuclide chelation [33,34].

Table 1 presents the chemical structures of all the homomultimeric FAPI ligands discussed in this review. From this overview, it is clear that, despite differences in the coupling, linker, and spacer chemistry, all FAP inhibitors are derived from UAMC1110. The final multimeric versions only differ in the position of their quinolinone part.

**Figure 2:**
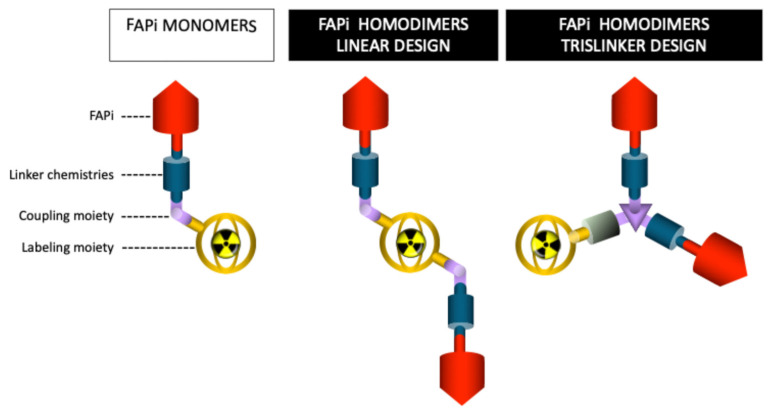
Variations in the design of radiotheranostic monomeric and homodimeric FAP inhibitors, illustrating linear and branching architectures.

Regarding the design of FAPI-based ligands, homodimeric designs generally adopt one of two architectures: linear or branching (Figure 2). Only a few linear dimers—specifically those with reported clinical data, such as DOTA/DOTAGA.(SA.FAPi)_2_ [18] and HBED-CC-FAPI-02/04 dimer [35]—have been developed. In this design, a single central chelator (e.g., DOTA or DOTAGA) connects two FAPI motifs in a linear chain. In contrast, the majority of dimeric derivatives feature a branching architecture. This design utilizes a central linker to connect the chelator to two individual FAPI-bearing sidechains in a “Y”-like form. (Because this central unit offers coupling opportunities in three directions, it is referred to as “tris-linker.”) These tris-linkers—often composed of amino acids (e.g., Glu, Lys, Asp, and Pro) or pyrrolidine—are engineered to balance the molecular flexibility, hydrophilicity, and the overall pharmacokinetics [36]. Typically, coupling moieties are inserted between chelator and the other components of the molecule, including spacer units. For example, to counteract the increased hydrophobicity introduced by multiple aromatic FAPI units, short polyethylene glycol (PEG) chains are frequently incorporated into the spacer to enhance solubility and improve the pharmacokinetic profile of the derived FAPI-based homodimers. The length of the PEG chain directly modulates the physicochemical properties of the radioligand. Shorter PEG chains (e.g., PEG_2_) provide a modest increase in hydrophilicity, whereas longer PEG chains (e.g., PEG_4_, PEG_6_, and PEG_12_) more effectively shield the molecule from opsonization and renal reabsorption, thereby prolonging circulation time and potentially enhancing tumor accumulation. However, excessively long PEG chains may increase molecular weight and slow tissue penetration [37,38].

Multimeric variants (e.g., trimer, tetramers, hexamers, octamers such as TetraOncoFAP-DOTAGA, and DOTA-4P(FAPI)_4_) employ a central scaffold to conjugate multiple FAP-inhibiting motifs (3, 4, 6, or 8) to a single radionuclide-chelating complex. This design introduces significant structural challenges: the larger molecular weight and increased hydrophobicity can reduce tissue penetration and lead to higher liver uptake [39]. To address these issues, the linker systems play a crucial role. They often incorporate long hydrophilic PEG chains or dendritic structures to improve solubility and minimize nonspecific binding [40].

Dimeric constructs are engineered to enhance binding avidity through the controlled spatial arrangement of two binding sites. In contrast, the higher-order multimers aim to function as irreversible “super-binders,” capitalizing on the high local density of FAP within the tumor stroma to increase the likelihood of target engagement. Although multimers demonstrate exponentially increased avidity in vitro, their larger size may impede extravasation and compromise homogeneous tumor distribution in vivo [22]. Accordingly, dimeric structures currently offer the most favorable balance, combining improved tumor retention with suitable pharmacokinetic properties for clinical theranostic applications. Meanwhile, the in vivo performance of higher-order multimers remains an active area of ongoing research, and a promising trimer variant was recently reported [21].

**Table 1:** The overview of dimeric and multimeric FAPI ligands in molecular design.

Valency	Architecture	Compound	R_1_	Structure	Ref.
**Dimer**	linear	DOTA.(SA.FAPi)_2_	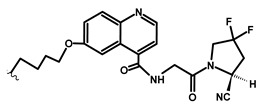	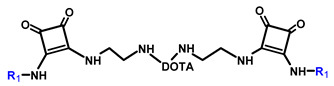	[18]
		DOTAGA.(SA.FAPi)_2_		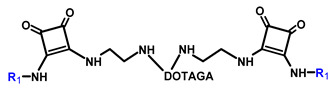	[18]
		HBED-CC-FAPI-02 dimer	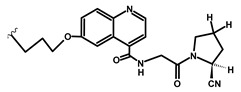	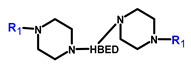	[35]
		HBED-CC-FAPI-04 dimer	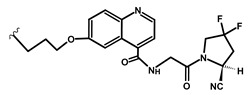		
	branching	DOTAGA.Glu.(FAPi)_2_	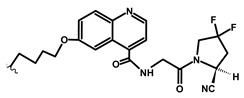	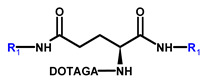	[41]
		DOTAGA.Npyr.(FAPi)_2_		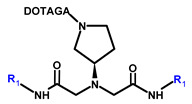	[42]
		NO3A.Glu.(FAPi)_2_		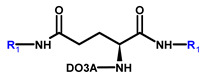	[41]
		NO3A.NPyr.(FAPi)_2_		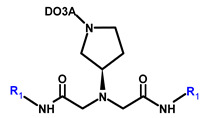	[42]
		BiOncoFAP-DOTAGA	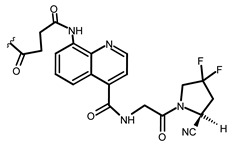	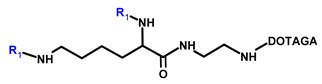	[20]
		DOTA-2P(FAPI)_2_	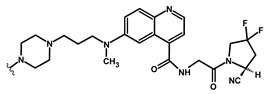	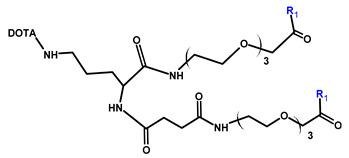	[43]
		ND-bisFAPI		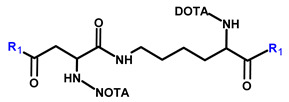	[44]
		FAPI-46-F1D		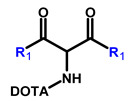	[45]
		DOTA-Suc-Lys-(FAPI-04)_2_	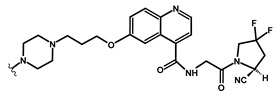	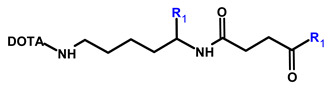	[46]
		DOTA/NOTA-E-(FAPI)_2_	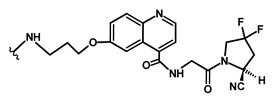	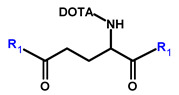	[47]
		LNC-1003		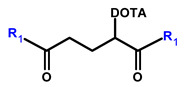	[48]
		HF_2_	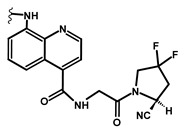	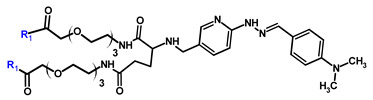	[49]
		L_2_	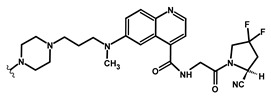	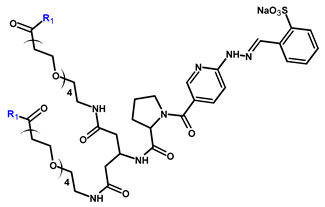	[50]
**Multimer**	dendritic	OncoFAP-23	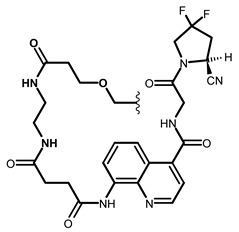	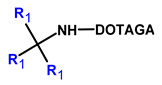	[23]
		tetraOncoFAP-DOTAGA		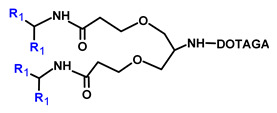	[23]
		DOTA/NOTA-4P(FAPI)_4_	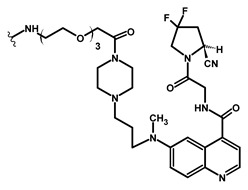	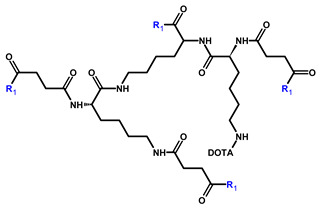	[22]
		HexaOncoFAP-DOTAGA	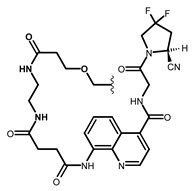	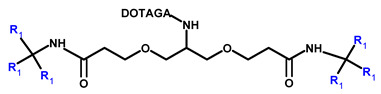	[23]
		OctaOncoFAP-DOTAGA		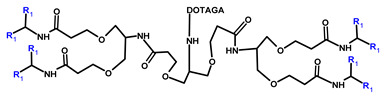	[23]

## 3. In Vitro Studies

***Radiolabeling and Stability***: Most FAPI-conjugated precursors listed in Table 2 were successfully labeled with ^68^Ga and/or ^177^Lu in some cases also with ^90^Y and ^225^Ac, achieving radiochemical yields exceeding 95%. The radiolabeling protocols are quite similar: the precursor is dissolved in the buffer (pH 3–6, commonly HEPES or NaOAc) and added to the radionuclide solution (^68^GaCl_3_ or ^177^LuCl_3_). The mixture is heated to >90 °C (typically 95 °C). The incubation time for ^68^Ga labeling is relatively short (10–15 min), while the ^177^Lu labeling requires a longer duration (20–30 min). An exception was the method by Hong et al., which utilized the acyclic chelator HBED instead of DOTA; this protocol achieved ^68^Ga labeling in sodium acetate buffer (pH 5.2) at room temperature [35]. Such instant kit-type FAPI-ligand labeling at room temperature procedures have been described earlier for DATA^5m^-conjugated derivatives, such as DATA^5m^.SA.FAPi [51] and AAZTA.SA.FAPi [52].

Following radiosynthesis, ^68^Ga and ^177^Lu-labelled radiotracers were purified and identified by HPLC and TLC. Subsequent stability studies in PBS, saline, and human or mouse serum demonstrated that the FAPI-dimer and -multimer constructs remained stable over periods of up to 7 days, comparable to their monomeric derivatives.

Two groups developed a kit-like method using tricine for ^99m^Tc radiolabeling with the FAPI-dimers HF_2_ and L_2_, enabling the diagnostic application of a FAPI-dimer tracer in single photon emission computed tomography (SPECT) [49,50].

***FAP Binding Affinity*** of these compounds was evaluated in vitro, a crucial step in identifying molecules with strong targeting potential for further thorough preclinical evaluation and subsequent clinical development. Two primary methods were used: (1) Enzyme activity assays utilize a FAP-specific substrate to directly measure catalytic cleavage and confirm the functional integrity of the enzyme’s active site, making it ideal for high-throughput inhibitor screening [53]. (2) Cell binding assays employ FAP-positive cells to assess a radioligand’s binding affinity and specificity, providing data on target engagement, internalization, and externalization that best predict in vivo performance [54]. The binding affinity was consistently reported as an IC_50_ value (Table 2). Most dimeric derivatives exhibited high affinity for FAP (IC_50_ = 0.1–1 nM). The affinity of compounds HF_2_ and L_2_, however, was approximately 10-fold lower. Notably, OncoFAP-DOTAGA derivatives (Philogen AG) were reported at exceptional affinity in the picomolar range, such as BiOncoFAP-DOTAGA and OncoFAP-11 with IC_50_ of 43 pM and 93 pM, respectively [23,55]. However, extremely high valency, as seen in Hexa- and OctaOncoFAP-DOTAGA, led to a substantial loss of potency due to steric hindrance, which impeded effective interaction with the FAP binding pocket [23]. A comparable decline in binding affinity was also observed for the tetravalent derivative DOTA-4P(FAPI)_4_, which showed a higher IC_50_ than its bivalent analogue, DOTA-2P(FAPI)_2_ [22].

Studies by both the Mainz University group and Philogen AG confirmed that the complexation with the corresponding cold metals had almost no effect on the high FAP-binding affinity of the ligands, as demonstrated by a comparison of IC_50_ values between precursors and their cold, with either gallium or lutetium, complexes [18,20,23,41,56].

***Internalization*** and ***Efflux*** assays are pivotal in the development of targeted radiopharmaceuticals, as they directly measure the key determinants of tumor uptake: cellular retention and persistence [57]. Internalization is very helpful for radiometal drugs (e.g., with ^68^Ga and ^64^Cu). These metals get trapped inside the cell’s lysosomes after entering, which helps keep the drug inside the tumor and stops it from leaking back out [58,59]. These assays are performed to quantify the rates of cellular uptake and subsequent release of the radiopharmaceutical, thereby characterizing its pharmacokinetic profile at the cellular level. This data is essential for selecting candidates with optimal in vivo retention for effective imaging or therapy. In a typical assay, cells expressing FAP are incubated with the FAPI-radiopharmaceutical sample. This sample can be taken directly from the reaction media or from the contents of lysed cells. The internalized/efflux radioactivity is then measured via gamma counting at predetermined time points. When required, a low-pH buffer wash is used to remove surface-bound drug, isolating the truly internalized fraction. Alternative methods, such as fluorescence microscopy or flow cytometry using labeled compounds, can also visualize and quantify cellular uptake dynamically over time [3,60]. The cellular uptake and efflux properties of these compounds have been characterized by only a limited number of research groups. In general, dimeric constructs have shown rapid cellular uptake comparable to their monomeric counterparts, with increasing amounts over the observation period. Furthermore, the internalization of FAPI ligands can be significantly inhibited by the addition of a competitive agent, confirming that the observed uptake is both specific and mediated by FAP [20,35,45,48,49]. This line of research has further demonstrated that the internalized dimeric ligands are also retained more effectively. Specifically, work by Läppchen et al. and Li et al. confirmed that the efflux rate of dimeric FAPI ligands is slower than that of their monomeric counterparts [44,56]. This reduced efflux directly contributes to a prolonged cellular retention time for the dimeric constructs.

The ***Lipophilicity*** of a drug compound is a critical property that profoundly influences its pharmacokinetic profile, including absorption, distribution, metabolism, and excretion (ADME). It directly influences membrane permeability, plasma protein binding, and the ability to cross biological barriers, such as the blood–brain barrier [61,62]. Achieving an optimal lipophilicity balance is therefore essential for ensuring adequate bioavailability and minimizing potential toxicity. This parameter is a key consideration in drug design, enabling researchers to predict in vivo performance and optimize lead compounds. The standard method for measuring lipophilicity is the shake-flask technique, which quantifies a compound’s partitioning between immiscible phases, typically n-octanol and a buffer such as phosphate-buffered saline. The result is expressed as the partition coefficient, logP (for neutral compounds), or the distribution coefficient, logD, at a specific pH (usually 7.4), which accounts for ionization [63,64]. As shown in Table 2, all di- and multimeric FAPI-radioligands are relatively hydrophilic with logD_7.4_ < −1, which suggests that the compounds have a small volume of distribution but possibly rapid renal clearance [65]. Subsequent in vivo distribution studies confirmed this prediction, demonstrating that the accumulation of these FAPI-based radiopharmaceuticals in kidneys is not critical.

**Table 2:** IC_50_ values of the homomultimeric FAPI probes against FAP with or without radiolabeling and their logD values (pH = 7.4).

	Precursor	IC_50_/nM	Cold-/Radiolabeling	IC_50_/nM	LogD_7.4_	Ref.
**Dimeric**	DOTA. (SA.FAPi)_2_	0.78 ^a^	^nat^Ga	1.05 ^a^	-	[18]
	DOTAGA.(SA.FAPi)_2_		^nat^Ga	0.90 ^a^	−2.02	[18]
		0.92 ^a^	^177^Lu	1.54 ^a^	−1.71	[56]
	DOTAGA.Glu.(FAPi)_2_		^68^Ga	-	−2.48	[41]
		0.26 ^a^	^177^Lu	0.33 ^a^	−2.77	
	DOTAGA.Glu_2_.(FAPi)_2_	0.48 ^a^	^68^Ga	-	−2.46	[42]
			^177^Lu		−2.71	
	DOTAGA.PEG_2_.Glu.(FAPi)_2_	0.37 ^a^	^68^Ga	-	−2.54	[42]
			^177^Lu		−2.48	
	DOTAGA.NPyr.(FAPi)_2_	0.50 ^a^	^68^Ga	-	−2.52	[42]
			^177^Lu		−2.57	
			^68^Ga	-	−2.08	[41]
	DO3A.Glu.(FAPi)_2_	0.26 ^a^	^177^Lu	-	−1.77	
	DO3A.Glu_2_.(FAPi)_2_	0.31 ^a^	^68^Ga	-	−2.59	[42]
	DO3A.NPyr.(FAPi)_2_	0.48 ^a^	^68^Ga	-	−2.43	[42]
			^177^Lu	-	−2.31	
	BiOncoFAP-DOTAGA	0.17 ^a^	^nat^Lu	0.19 ^a^	−3.60	[20]
	BiOncoFAP-DOTAGA	0.093 ^a^	^nat^Lu	0.107 ^a^	−3.75	[23]
	BiOncoFAP-11	0.043 ^a^	^177^Lu	-	-	[55]
	DOTA-2P(FAPI)_2_	-	^68^Ga	2.06 ^b^	-	[43]
			^177^Lu	-	-	
	HF_2_	9.37 ^b^	^99m^Tc-TPPTS	-	−2.64	[49]
			^99m^Tc-EDDA	-	−1.46	
			^99m^Tc-TPPM	-	−0.79	
	L_2_	8.41 ^a^	^99m^Tc-L2-TPPTS	-	−3.01	[50]
			^99m^Tc-L2-TPPMS	-	−1.71	
			^99m^Tc-L2-PDA	-	−3.09	
			^99m^Tc-L2-NIC	-	−3.05	
	LNC1013	-	^68^Ga	7.15 ^b^	−2.23	[48]
	FAPI-3	-	^68^Ga	6.5 ^b^	−2.16	[48]
	FAPI-5	-	^68^Ga	6.3 ^b^	−2.08	[48]
	DOTA-Suc-Lys-(FAPI-04)_2_	-	^68^Ga	-	−3.05	[46]
			^177^Lu	-	−3.05	
	HBED-CC-FAPI-02 dimer	-	^68^Ga	4.99 ^b^		[35]
	HBED-CC-FAPI-04 dimer	-	^68^Ga	5.10 ^b^		[35]
	ND-bisFAPI	0.25 ^b^	^177^Lu	-	−2.55	[44]
	NOTA-E-(FAPI)_2_	0.47 ^a^	-	-	-	[47]
	DOTA-E-(FAPI)_2_	0.16 ^a^	-	-	-	[47]
	FAPI-46-F1D	0.16 ^a^	^177^Lu	-	−2.28	[45]
**Trimeric**	OncoFAP-23	0.013 ^a^	^nat^Lu	0.012 ^a^	−3.57	[23]
**Tetrameric**	TetraOncoFAP-DOTAGA	0.002 ^a^	^nat^Lu	0.003 ^a^	−3.11	[23]
	DOTA-4P(FAPI)_4_	15.56 ^b^	^68^Ga	-	-	[22]
	NOTA-4P(FAPI)_4_	16.27 ^b^	^64^Gu	-	-	[22]
**Hexametric**	HexaOncoFAP-DOTAGA	20 ^a^	^nat^Lu	22 ^a^	−2.55	[23]
**Octameric**	OctaOncoFAP-DOTAGA	26 ^a^	^nat^Lu	29 ^a^	−2.32	[23]

^a^ Measurement of IC_50_ values by enzyme assays. ^b^ Measurement of IC_50_ values by cell binding experiments.

The ***Selectivity Assay*** is also a non-negotiable prerequisite for developing a safe and effective targeted radiopharmaceutical. Poor selectivity can lead to severe off-target toxicity, high background uptake leading to false positives in imaging, radiation damage to healthy organs, and a narrow therapeutic window [66,67]. As FAP belongs to the S9b serine protease family, which includes dipeptidyl peptidase 4 (DPP4), DDP8, and DDP9 and prolyl endopeptidase (PREP) [68], selectivity over these homologs is essential. Researchers used substrate-competition enzymatic assays to evaluate competitive binding to the mentioned serine proteases that are related to FAP. The Mainz University group reported that branched FAPI ligands exhibited superior binding selectivity compared to linear derivatives [41]. Similar to their high affinity, the multimeric OncoFAP-DOTAGA derivates also showed excellent FAP selectivity [23]. Interestingly, the ligands developed by Mainz University group demonstrated superior selectivity for FAP over PREP and DPP4, whereas the compounds from Philogen AG were more selective for FAP over DPP8 and DPP9 (Table 3).

**Table 3:** IC_50_ values and selectivity index (*SI*) of the FAPI ligands against FAP and the related serine proteases (PREP, DDP4, DDP8, and DPP9).

	Compound	FAP/nM	PREP/µM	DPP4/µM	DPP8/µM	DPP9/µM	*SI* (PREP/FAP)	*SI* (DPP4/FAP)	*SI* (DPP8/FAP)	*SI* (DPP9/FAP)	Ref.
**Monomeric**	OncoFAP-DOTAGA	0.52	2.90	>10	>10	6.30	5577	>19,231	>19,231	12,115	[23]
**Dimeric**	DOTA.(SA.FAPi)_2_	0.78	0.42	0.44	1.33	0.96	538	564	1705	1231	[18]
	DOTAGA.(SA.FAPi)_2_	0.92	0.39	0.4	0.42	0.16	424	435	457	174	[18]
	DOTAGA.Glu.(FAPi)_2_	0.26	0.59	1.19	0.029	0.083	2269	4577	112	319	[41]
	DOTAGA.Glu_2_.(FAPi)_2_	0.48	0.63	1.37	0.041	0.23	1306	2854	84	478	[42]
	DOTAGA.PEG_2_.Glu.(FAPi)_2_	0.37	0.50	2.44	0.023	0.059	1351	6581	63	159	[42]
	DOTAGA.NPyr.(FAPi)_2_	0.50	0.73	2.68	0.106	0.35	1454	5312	211	702	[42]
	DO3A.Glu.(FAPi)_2_	0.60	1.00	0.54	1.03	0.95	1667	900	1717	1583	[41]
	DO3A.Glu_2_.(FAPi)_2_	0.31	0.41	1.03	0.074	0.13	1308	3288	237	415	[42]
	DO3A.NPyr.(FAPi)_2_	0.48	1.86	3.9	0.208	0.2	3899	8187	437	417	[42]
	BiOncoFAP-DOTAGA	0.093	0.302	>10	>10	0.372	3247	>107,527	>107,527	4000	[23]
**Trimeric**	TriOncoFAP-DOTAGA	0.014	0.011	>10	3.10	0.015	786	>714,286	221,429	1071	[23]
**Reference**	UAMC-1110 ^a^	0.43	1.80	>10	>10	4.70	4186	>23,256	>23,256	10,930	[18]

^a^ IC_50_ values of UAMC-1110 were adapted from the publication of Mainz University group because the IC_50_ values of UAMC-1110 for FAP and DPP9 in the original publication [69] of University of Antwerp group were determined with non-human protein in enzyme assays.

## 4. Quantitative Biodistribution in Tumor-Bearing Mice

The quantitative biodistribution data presented in Table 4 and Tables S1–S4 provide valuable insights into the pharmacokinetics and tumor-targeting efficiency of various FAPI-conjugated radiopharmaceuticals across different animal models. These tables summarize key metrics—including tumor accumulation, tumor-to-liver, tumor-to-kidney, tumor-to-blood, and tumor-to-heart ratios at multiple time points post-injection—offering a comprehensive overview of the in vivo behavior of these compounds.

Tumor models were established by implanting cell lines expressing human or murine fibroblast activation protein (hFAP) into mice, presenting a range of cancer types, including non-small cell lung cancer, fibrosarcoma, prostate adenocarcinoma, glioblastoma, renal cell carcinoma, ovarian carcinoma, and epidermoid carcinoma [10,70]. Notably, the xenografts used in these studies differ in origin and consequently in FAP expression patterns: **(a) Wild-type cell-derived xenografts**—These models originate from non-engineered cells and exhibit a mixture of murine and human FAP expressed in the tumor stroma, mimicking the natural setting. Examples include studies by Läppchen et al. [56] and Zhong et al. [46]. **(b) Transfected cell line-derived xenografts**—In these models, hFAP is overexpressed on the surface of tumor cells rather than in the stroma. The engineered hFAP retains enzymatic activity and various biological functions, including internalization and promotion of proliferation, migration, and invasion. This type of xenograft has been widely used, as transfection ensures stable, high-level FAP expression, unlike the often lower basal expression in parental cell lines [71]. **(c) Patient-derived xenografts (PDX)**—These originate from patient tissue and initially express hFAP, but after several passages may also show murine FAP expression in the stroma. Chen’s research group is notable for employing PDX models in FAP-targeted diagnosis and therapy [43,72]. Although murine FAP expression was not explicitly confirmed in their studies, the PDX establishment followed their previously described protocol for non-small cell lung cancer models, in which murine integrin expression was detected in both integrin α_v_β_3_-positive and -negative PDX tumors [73]. Moreover, PDX models are valued for their ability to closely recapitulate original tumor heterogeneity, preserve cell–cell interactions, and maintain the tumor microenvironment [74]. In all of the preclinical studies reviewed herein and summarized in Table 4 and Tables S1–S4, the radiolabeled FAPI homomultimers were administered intravenously (IV), which is the standard route for radiotracer administration in both preclinical pharmacokinetic and therapeutic efficacy studies.

Compared to their monomeric counterparts, dimeric FAPI derivatives exhibited a clear trend toward significantly prolonged tumor retention, as summarized in Table 4. While monomeric FAPI ligands were nearly cleared from tumors within 24 h post-injection (p.i.), the dimeric constructs maintained high accumulation levels over this period. Across studies, multimeric ligands generally exhibited enhanced tumor uptake, with the highest levels of accumulation and longest retention times being associated with trimeric and tetrameric constructs. In contrast, further increases in valency to hexameric and octameric structures correlated with a sharp reduction in tumor residency. This diminished retention aligns with their reported decrease in binding affinity, underscoring that optimal pharmacokinetic performance depends on balancing multimerization with preserved target engagement.

**Figure 3:**
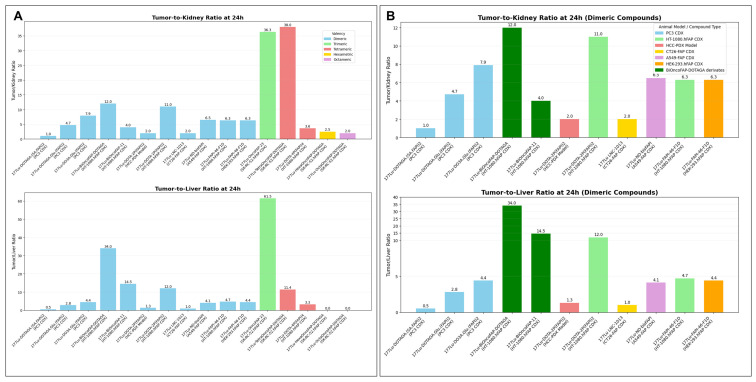
Tumor-to-kidney (T/K) and tumor-to-liver (T/L) ratios for various ^177^Lu labeled-FAPI ligands in animal models. Panel (**A**) T/K and T/L ratios of dimeric and multimeric FAPI radioligands at 24 h p.i. Panel (**B**) T/K and T/L ratios of dimeric FAPI radioligands at 24 h p.i.

**Figure 4:**
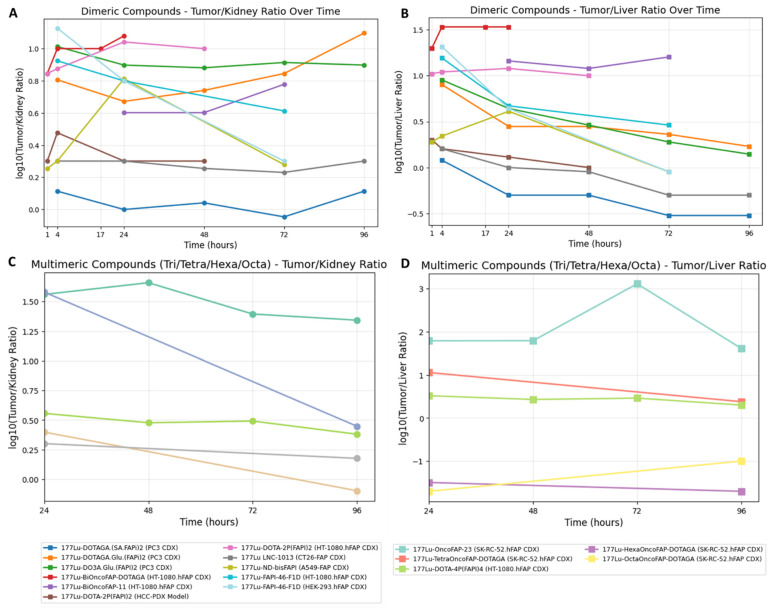
Tumor-to-kidney (T/K) and tumor-to-liver (T/L) ratios for various ^177^Lu-labeled FAPI ligands in animal models; logarithmic tumor/kidney (**A**) and tumor/liver (**B**) ratios of dimeric FAPI radioligands at 1, 4, 24, 48, 72, and 96 h p.i. logarithmic tumor/kidney (**C**) and tumor/liver (**D**) ratios of multimeric FAPI radioligands at 24, 48, 72, and 96 h p.i.

While the dimeric and multimeric FAPI-radioligands achieved high tumor uptake up to 24 h p.i., they also demonstrated rapid clearance from healthy tissues. This was reflected in rising tumor-to-blood (Table S3) and tumor-to-heart ratios (Table S4) over time, indicating fast clearance from the blood pool. To assess long-term pharmacokinetics, tumor-to-liver (T/L) and tumor-to-kidney (T/K) uptake radios were compared for a series of ^177^Lu-labeled FAPI ligands, including [^177^Lu]Lu-DOTAGA.(SA.FAPi)_2_, [^177^Lu]Lu-DOTAGA.Glu.(FAPi)_2_, [^177^Lu]Lu-DO3A.Glu.(FAPI)_2_, [^177^Lu]Lu-BioncoFAP-DOTAGA, [^177^Lu]Lu-BiOncoFAP-11, [^177^Lu]Lu-DOTA-2P(FAPI)_2_, [^177^Lu]Lu-LNC-1013, [^177^Lu]Lu-ND-bisFAPI, [^177^Lu]Lu-FAPI-46-F1D, [^177^Lu]Lu-OncoFAP-23, [^177^Lu]Lu-TetraOncoFAP-DOTAGA, [^177^Lu]Lu-DOTA-4P(FAPI)_4_, [^177^Lu]Lu-HexaOncoFAP-DOTAGA, and [^177^Lu]Lu-OctaOncoFAP-DOTAGA (Figure 3 and Figure 4). The pharmacokinetic profiles of dimeric constructs varied considerably, with T/L (1–34) and T/K ratios (1–12) spanning a wide range; these ratios generally decreased after 24 h for most compounds. In contrast, certain multimeric ligands exhibited superior and more sustained targeting properties compared to the dimeric group.

Among the series, OncoFAP-DOTAGA derivates demonstrated distinctive behaviors: for instance, the trimeric construct [^177^Lu]Lu-OncoFAP-23 showed exceptional and prolonged target engagement in the SK-RC-52.hFAP model, achieving a remarkable T/L ratio of ~61.5 at 24 h that increased dramatically to >1200 by 72 h. The dimeric derivates [^177^Lu]Lu-OncoFAP-23 showed stable T/L ratios and increasing T/K ratios over time in the same animal model. Conversely, higher-order multimers, such as hexameric and octameric constructs, exhibited significantly diminished efficacy, with near-zero T/L ratios, indicating a complete lack of effective tumor targeting (Figure 4).

Importantly, T/L and T/K ratios were influenced by the choice of animal model, underscoring the impact of the biological context. For example, the performance of [^177^Lu]Lu-DOTA-2P(FAPI)_2_ varied significantly between HT-1080.hFAP CDX and HCC-PDX models (Figure 4), highlighting how factors such as tumor type and FAP expression levels can affect experimental outcomes.

**Table 4:** Biodistribution data of different FAPI homomultimers for tumors in animal models at 1, 2, 3, 4, 8, 24, 48, 72, and 96 h post-injection. The data were calculated as mean of the % injected activity per gram of tissue (%I.A./g).

	Precursor	Labeling	Animal Model	Tumor %I.A./g									
				1 h	2 h	3 h	4 h	8 h	24 h	48 h	72 h	96 h	Ref
Mono-meric	FAPI-04	^177^Lu	A549-FAP CDX	19.7	-	-	12.3	-	5.0	-	1.6	-	[44]
	FAPI-46	^177^Lu	HT-1080.hFAP CDX	-	-	-	9.6	-	2.7	-	1.6	-	[45]
			HEK-293 CDX	-	-	-	10.3	-	3.4	-	0.4	-	
	DOTA.SA.FAPi	^68^Ga	PC3 CDX	6.1	5.4	5.0	-	-	-	-	-	-	[56]
		^68^Ga	U87MG CDX	6.5	5.2	4.9	-	-	-	-	-	-	
		^177^Lu	PC3 CDX	9.6	-	-	8.9	-	1.8	1.0	-	-	
	OncoFAP-DOTAGA	^177^Lu	HT-1080.hFAP CDX	18.5 ^a^	-	-	19 ^a^	-	4 ^a^				[20]
Di-meric	DOTAGA. (SA.FAPi)_2_	^68^Ga	PC3 CDX	6.3	6.1	6.3	-	-	-	-	-	-	[56]
		^68^Ga	U87MG CDX	7.1	9.0	9.4	-	-	-	-	-	-	
		^177^Lu	PC3 CDX	-	-	-	8.6	-	5.6	3.9	2.6	1.8	
	DOTAGA.Glu. (FAPi)_2_	^68^Ga	PC3 CDX	16.0 ^a^	17.5 ^a^	18.0 ^a^	-	-	-	-	-	-	[41]
		^177^Lu	PC3 CDX	-	-	-	16.0 ^a^	-	7.0 ^a^	5.5 ^a^	3.5 ^a^	2.5 ^a^	
	DO3A.Glu. (FAPI)_2_	^68^Ga	PC3 CDX	19.0	19.6	17.5	-	-	-	-	-	-	[75]
		^177^Lu	PC3 CDX	-	-	-	15.0	-	7.5	3.1	1.4	1.0	
	BiOncoFAP-DOTAGA	^177^Lu	HT-1080.hFAP CDX	28.0 ^a^	-	-	32.0 ^a^	-	19.0 ^a^	-	-	7.0 ^a^	[20]
	BiOncoFAP-11	^177^Lu	HT-1080.hFAP CDX	-	-	-	-	-	23.0	16.3	14.5	-	[55]
	DOTA-2P(FAPI)_2_	^68^Ga	Hepatocellular carcinoma (HCC)-PDX	9.0	-	-	7.2	-	-	-	-	-	[43]
		^177^Lu	HCC-PDX	6.0 ^a^	-	-	8.3	-	4.0 ^a^	2.9	-	-	[72]
		^177^Lu	HT-1080.hFAP CDX	20.8 ^a^	-	-	21.0 ^a^	-	21.0 ^a^	19.7	-	-	[72]
	HF_2_	^99m^Tc-TPPTS	HT-1080.hFAP CDX	40.1	39.0	-	35.8	-	-	-	-	-	[49]
			U87MG CDX	12.5	22.9	-	12.0	-	-	-	-	-	
	L_2_	^99m^Tc-L2-TPPTS	U87MG CDX	22.0	-	-	16.7	-	-	-	-	-	[50]
		^99m^Tc-L2-PDA	U87-MG CDX	26.6	-	-	18.3	-	-	-	-	-	
	LNC1013	^68^Ga	HT-1080.hFAP CDX	14.0 ^a^	12.0 ^a^	-	7.0 ^a^	-	-	-	-	-	[48]
		^177^Lu	CT26-FAP CDX	-	-	-	4.2	-	1.8	0.8	0.3	0.2	[76]
	FAPI-3	^68^Ga	HT-1080.hFAP CDX	13 ^a^	11 ^a^	-	6.0 ^a^	-	-	-	-	-	[48]
	FAPI-5	^68^Ga	HT-1080.hFAP CDX	13 ^a^	8 ^a^	-	6.0 ^a^	-	-	-	-	-	[48]
	DOTA-Suc-Lys-(FAPI-04)_2_	^68^Ga	SKOV3 CDX	7.0 ^a^	-	6.8	-	-	-	-	-	-	[46]
			A431 CDX	6.0 ^a^	-	6.2	-	-	-	-	-	-	
			H1299 CDX	6.8 ^a^	-	7.2	-	-	-	-	-	-	
	HBED-CC-FAPI-02 dimer	^68^Ga	U87MG tumor (FAP+) CDX	25.4	16.7	-	-	-	-	-	-	-	[35]
	ND-bisFAPI	^18^F	A549-FAP CDX	16.0 ^a^	15.4 ^a^	15.0 ^a^	15.5 ^a^	-	-	-	-	-	[44]
		^177^Lu	A549-FAP CDX	14.3	-	-	14.7	-	32.6	-	3.7	-	
	NOTA-E-(FAPI)_2_	^18^F	U87MG CDX	15.9	17.3	17.6	-	-	-	-	-	-	[47]
	DOTA-E-(FAPI)_2_	^64^Cu	U87MG CDX	22.7	23.0	-	-	-	-	-	-	-	[47]
		^68^Ga	U87MG CDX	15.9	16.2	16.9	-	-	-	-	-	-	
	FAPI-46-F1D	^177^Lu	HT-1080.hFAP CDX	-	-	-	10.5	-	6.4	-	3.6	-	[45]
		^177^Lu	HEK-293.hFAP CDX	-	-	-	17.2	-	5.9	-	1.4	-	
Tri-meric	OncoFAP-23	^177^Lu	SK-RC-52.hFAP CDX	-	-	-	-	-	41.7	28.1	20.2	16.3	[21]
Tetra-meric	TetraOncoFAP-DOTAGA	^177^Lu	SK-RC-52.hFAP CDX	-	-	-	-	-	40.0 ^a^	-	-	17.0 ^a^	[23]
	DOTA-4P(FAPI)_4_	^177^Lu	HT-1080.hFAP CDX	-	-	-	-	-	21.4	19.2	18.8	14.8	[22]
Hexa-metric	HexaOncoFAP-DOTAGA	^177^Lu	SK-RC-52.hFAP CDX	-	-	-	-	-	2.0	-	-	1.0 ^a^	[23]
Octa-meric	OctaOncoFAP-DOTAGA	^177^Lu	SK-RC-52.hFAP CDX	-	-	-	-	-	1.0	-	-	1.5 ^a^	[23]

^a^ values from references were read from the graphs and therefore do not indicate precise values.

## 5. Radiotherapy In Vivo Xenograft Models

Following the promising preclinical and clinical data on FAPI-targeted radiotracers for tumor imaging, considerable efforts have been directed toward evaluating their potential as therapeutic agents. The development of FAPI-based radioligands labeled with therapeutic radionuclides, such as ^177^Lu, has opened new avenues for selectively delivering cytotoxic radiation to the tumor stroma, in particular, to FAP-expressing CAFs. These approaches aim to disrupt the supportive tumor microenvironment, reduce tumor growth, and potentially enhance the efficacy of other antitumor treatments. Early investigations have primarily focused on assessing the therapeutic performance of monomeric and multimeric FAPI radioligands in preclinical models, providing valuable insights into their pharmacokinetics, tumor uptake, and antitumor efficacy (Table 5).

Compared to their monovalent counterparts, homomultimeric FAPI-radioligands demonstrated significantly improved antitumor efficacy [20,44,72]. This anticancer effect was dose-dependent. For instance, a single administration of ^177^Lu-(FAPI-04)_2_ at a dose of 14.8 MBq/mouse resulted in superior antitumor activity compared to 7.4 MBq/mouse in human A431 tumor-bearing mice [46]. Similarly, [^177^Lu]Lu-OncoFAP-23 showed optimal outcomes at 30 MBq/mouse compared to doses of 5 and 15 MBq/mouse [21]. However, [^177^Lu]Lu-ND-bisFAPI exhibited an opposite trend, in that the survival rate was slightly worse in the high dose group than the low dose group (27 days vs. 37 days) [44]. In addition, the therapeutic efficacy varied across experimental models. In the HT-1080-FAP xenografts with extremely high FAP expression, [^177^Lu]Lu-DOTA-4P(FAPI)_4_ and [^177^Lu]Lu-DOTA-2P(FAPI)_2_ both rapidly and potently inhibited tumor growth, with no significant difference observed between them. In contrast, in U87MG tumor xenografts with moderate or mild FAP expression, ^177^Lu-DOTA-4P(FAPI)_4_ showed significantly better antitumor efficacy at the same radioactivity dosage than [^177^Lu]Lu-DOTA-2P(FAPI)_2_ and [^177^Lu]Lu-FAPI-46 did [22]. A similar difference was also observed in a comparative study using CT-26.hFAP xenografts (low FAP expression level) and SK-RC-52.hFAP xenografts (high FAP expression level) treated with a combination of [^177^Lu]Lu-OncoFAP-23 plus L19-IL2. The same treatment protocol induced a better therapeutic outcome in the high-FAP model than in the low-FAP model, with complete remission rates of 4/4 versus 3/4, respectively [21].

The above studies clearly demonstrate that the therapeutic performance of FAPI-targeted radioligands has been influenced by the dosing regimen, particularly regarding single versus fractionated administrations. Most preclinical studies to date have employed single-dose protocols to facilitate straightforward assessment of dose-response relationships and radioligand biodistribution. However, while a single high-activity injection can achieve strong initial tumor control, it may also increase the risk of acute radiation exposure to healthy organs such as the kidneys and bone marrow [77,78]. In contrast, fractionated or repeated administrations could provide a more favorable therapeutic index by allowing cumulative tumor irradiation while permitting normal tissue recovery between cycles. Although direct comparative data between single and fractionated FAP-radioligand treatments remain limited, available evidence suggests that optimized fractionation schemes could further enhance therapeutic outcomes, particularly in tumors with moderate or heterogeneous FAP expression [79,80]. Future studies assessing the kinetics of tumor repopulation, radioligand retention, and normal tissue repair following sequential administrations will be essential for defining the most effective and safe dosing strategy [81,82,83].

**Table 5:** Summary of radiotherapeutic efficacy with different therapeutic FAPI-based radioligands.

Compound	Model	Tumor Type	Level of FAP Expression on Tumors	Therapeutic Doses (MBq/Mouse)	Number of Doses	Combi-Therapy	Results	Ref.
[^177^Lu]Lu-DOTAGA.(SA.FAPi)_2_	4T1 murine TNBC model	mammary carcinoma	high	18.5	2	No	significant tumor inhibition, median survival = 30 days	[84]
				18.5	2	Olaparib (2 × 50 mg/kg)	significant tumor inhibition, median survival = 35 days	
	4T1 murine TNBC model	mammary carcinoma	high	18.5	2	No	significant tumor inhibition, median survival = 35 days	[85]
				18.5	2	AMD3100 (daily 5 mg/kg for 2 weeks)	significant tumor inhibition, median survival = 45 days	
[^177^Lu]Lu-BiOncoFAP-DOTAGA	HT-1080.hFAP CDX	fibrosarcoma	high	15	1	No	selective and potent antitumor activity, no measurement of survival	[20]
				70	1	No		
	SK-RC-52.hFAP CDX	renal carcinoma	high	5	1	No	tumor inhibition, no measurement of survival	[21]
[^177^Lu]Lu-(FAPI-04)_2_	human A431 CDX_2_	Squamous cell carcinoma	n.s.	7.4	1	No	tumor inhibition, no measurement of survival	[46]
				14.8	1	No	best antitumor efficacy, no measurement of survival	
				7.4	2	No	best antitumor efficacy, no measurement of survival	
[^177^Lu]Lu-ND-bisFAPI	A549-FAP CDX	lung carcinoma	high	18.5	1	No	median survival = 37 days	[44]
				37	1	No	median survival = 27 days	
[^177^Lu]Lu-DOTA-2P(FAPI)_2_	HCC-PDX	hepatocellular carcinoma	high	29.6	1	No	significant tumor inhibition, no measurement of survival	[72]
	HT-1080.hFAP CDX	fibrosarcoma	high	18.5	1	No	6 of 6 complete response, no recurrence	[72]
				29.6	1	No		
	U87MG CDX	glioblastoma	moderate	29.6	1	No	better tumor inhibition, mice (6/6) lived until the end of the experiment (22 days) and median survival time not reached	[22]
[^177^Lu]Lu-OncoFAP-23	SK-RC-52.hFAP CDX	renal carcinoma	high	5	1	No	Antitumor inhibition, no measurement of survival	[21]
				15	1	No	1 of 4 complete remissions	
				30	1	No	2 of 4 complete remissions	
				5	1	L19-IL2 (3 × 2.5 mg/kg)	4 of 4 complete remissions	
	CT-26.hFAP CDX	colorectal carcinoma	low	5	1	L19-IL2 (3 × 2.5 mg/kg)	3 of 4 complete remissions	[21]
[^177^Lu]Lu-DOTA-4P(FAPI)_4_	HT-1080.hFAP CDX	fibrosarcoma	high	29.6	1	No	best tumor inhibition, mice (6/6) lived until the end of the experiment (22 days) and median survival time not reached	[22]
	U87MG CDX	glioblastoma	moderate	29.6	1	No		

## 6. Combination Radiotherapy In Vivo Xenograft Models

While these findings clearly demonstrate the promising therapeutic potential of FAP-targeted radioligands as monotherapies, several studies have also highlighted their limitations, particularly in heterogeneous tumor environments or at suboptimal dosing levels [86,87]. To further enhance therapeutic efficacy and overcome resistance mechanisms, recent research has focused on integrating FAP-radioligand therapy with other treatment modalities [88,89,90]. Such combination strategies aim to exploit synergistic effects between FAP-targeted radiation delivery and complementary agents that modulate DNA repair, immune activation, or tumor microenvironment remodeling.

One compelling line of research is exemplified by the work of Bao et al., who investigated this approach using [^177^Lu]Lu-DOTAGA.(SA.FAPi)_2_ in combination with olaparib or AMD3100 in a 4T1 murine triple-negative breast cancer xenograft model. Their study demonstrated that both [^177^Lu]Lu-DOTAGA.(SA.FAPi)_2_ alone and in combination with 50 mg/kg olaparib resulted in significantly greater tumor suppression than olaparib alone. Notably, the combination group exhibited the longest median survival of all groups [84]. Similarly, the administration of [^177^Lu]Lu-DOTAGA.(SA.FAPi)_2_, either alone or combined with AMD3100, showed more potent antitumor activity than AMD3100 alone. The median survival time for the combination treatment group exceeded 45 days [85]. Impressively, subsequent toxicity analyses confirmed that treatment with [^177^Lu]Lu-DOTAGA.(SA.FAPi), both alone and in combination, induced no damage to the major organs, such as kidney, liver, etc.

Galbiati et al. also investigated the combination of [^177^Lu]Lu-OncoFAP-23 and L19-IL2 in CT-26.hFAP and SK-RC-52.hFAP mouse models. In both xenograft models, the combination treatment significantly improved the therapeutic index and demonstrated strong anticancer efficacy. Further optimization studies, which varied the timing (every 2 or 5 d) and number (1 or 3) of L19-IL2 doses, revealed that therapeutic efficacy was independent of the interval between the three times of L19-IL2 administrations. However, multiple administrations of L19-IL2 resulted in stronger antitumor activity, whereas a single injection of L19-IL2 in the combination treatment led to complete remissions in only one of four mice. Subsequent toxicological studies of OncoFAP-23-DOTAGA and its cold-labeled analog, ^nat^Lu-OncoFAP-23, showed no clinically significant hematological and histopathological changes attributable to either compound [21]. However, the authors did not provide any safety data for combination treatment groups.

The encouraging outcomes of these preclinical studies highlight the potential of combination strategies to enhance the therapeutic benefits of FAP-targeted radioligand therapy. By integrating FAP-directed radiation with agents that modulate DNA repair pathways, immune activation, or tumor microenvironment dynamics, synergistic effects can be achieved that surpass those of single-agent treatments. For instance, the combination with PARP inhibitors, such as olaparib, enhances DNA damage persistence and apoptosis in tumor cells exposed to β-emission from ^177^Lu [91,92], while pairing with immunomodulators like L19-IL2 may stimulate immune-mediated tumor clearance and sustain antitumor responses [93,94]. Similarly, the addition of CXCR4 inhibitors such as AMD3100 can disrupt tumor-stroma interactions, facilitating improved radioligand penetration and tumor irradiation [95,96,97]. Most reported combination regimens demonstrated enhanced efficacy without additional systemic toxicity, suggesting a favorable safety margin and translational potential. Nevertheless, further studies are needed to optimize combination scheduling, dosing ratios, and treatment sequences, as well as to evaluate possible overlapping toxicities or immunologic interactions in more clinically relevant models. Establishing the mechanistic basis and therapeutic window of such multimodal approaches will be crucial for guiding their safe and effective translation into clinical trials.

## 7. Dose Escalation Studies

A key factor limiting the therapeutic window of FAP-targeted radioligands (TRLs) could be the elevated background uptake in non-target tissues, which could obscure diagnostic images and increase toxicity risks in therapy. To optimize the biodistribution profile—achieving maximal tumor uptake while minimizing healthy organ exposure—systematic mass dose escalation studies are essential [98,99].

These studies systematically vary the injected molar dose (the mass of the pharmaceutical, basically referred to as the mass of the precursor used) to identify the optimal quantity that saturates non-specific binding sites without flooding the system. This concept is well-established for other targets like PSMA and SSTR, where the molar activity (the ratio of radioactive to non-radioactive molecules) critically impacts tumor targeting and organ clearance [100,101]. The findings from dose escalation studies with multimeric FAPI-conjugates often reveal significant differences in the optimal injected mass between patient cohorts. These variations can be attributed to several factors. A primary consideration is the variable concentration of soluble FAP (sFAP) in human plasma, which acts as a circulating sink that competitively binds the radiopharmaceutical, diverting it from the tumor and altering its pharmacokinetics. Therefore, an optimal injected mass must be sufficient to overcome this sFAP “pool” to effectively target tumor-bound FAP [102].

Ultimately, the critical importance of identifying this optimal injected mass is proven in a therapeutic context. Therapy studies with FAP-targeted radioligands demonstrate that only at the optimal mass is a favorable tumor-to-background ratio achieved, which translates directly into enhanced therapeutic efficacy and a reduced risk of off-target radiation damage to healthy organs, thereby validating the precision required in dosing. Table 6 presents a summary of dose escalation studies with dimeric FAPI-radioligands.

For example, Zhu et al. demonstrated that a dose of 10 nmol/kg of [^177^Lu]Lu-DOTAGA.(SA.FAPi)_2_ resulted in superior tumor inhibition in 4T1 tumor-bearing mice compared to 50 nmol/kg at the same radioactivity dose [103]. Expanding on this, the Gourni group conducted dose escalation studies using ^68^Ga-labeled dimeric FAPI ligands in Table 6. Both biodistribution and PET imaging data indicated that injected doses of 350–600 pmol/mouse maximized tumor radioactivity accumulation. Based on mouse weights of 20–22 g at sacrifice, this corresponds to an optimal dose range of 16.7–28.6 nmol/kg [105]. Similarly, ^177^Lu-labeled OncoFAP variants exhibited an optimal dose range of 90–250 nmol/kg in SK-RC-52.hFAP tumor-bearing mice, achieving high tumor-to-organ ratios [104]. This finding suggests that OncoFAP-based radioligands require higher molar doses than [^177^Lu]Lu-DOTAGA.(SA.FAPi)_2_ to enhance tumor radiation accumulation.

Galbiati et al. further explored the effect of molar activity by administering a fixed molar doses of 250 nmol/kg [^177^Lu]Lu-OncoFAP-23 at two different activity levels (80 MBq/mg vs. 2 GBq/mg). They observed no significant differences in tumor or healthy organ uptake [104]. Together, these studies demonstrated that high molar doses of FAPI-radioligands can reduce tumor accumulation and tumor-to-organ ratios, likely due to receptor saturation by excess unlabeled compound in circulation. In contrast, variations in tumor burden and injected molar activity showed no significant impact on distribution profiles.

## 8. Clinical Applications

Building upon the encouraging preclinical data, it has been well established that multimerization of FAPI motifs can significantly prolong tumor retention and improve the overall pharmacokinetic behavior of FAPI-based radiopharmaceuticals in vivo. Based on these promising results, several multimeric FAPI constructs have advanced into clinical evaluation. To date, seven compounds have been investigated in human studies, highlighting an important step toward translating these agents into clinical practice (Table 7).

### 8.1. DOTAGA.(SA.FAPi)_2_ and DOTAGA.Glu.FAPi_2_

**Figure 5:**
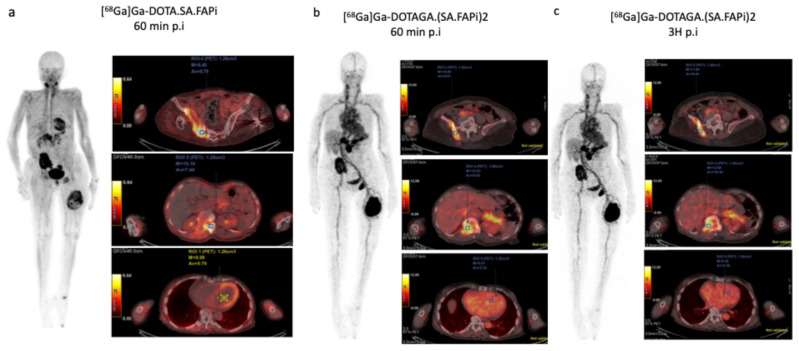
A 63-year-old woman with papillary thyroid carcinoma underwent FAPI PET/CT imaging. (**a**) Maximum-intensity projection (MIP) images of [^68^Ga]Ga-DOTA.SA.FAPi obtained at 60 min post-injection demonstrates physiological tracer uptake in the salivary glands, pancreas, and myocardium, along with multiple FAPI-avid skeletal lesions involving the right ilium, left ischial tuberosity, and left femur. (**b**,**c**) MIP images acquired using [^68^Ga]Ga-DOTAGA.(SA.FAPi)_2_ at 60 min and 3 h post-injection, respectively, show physiological biodistribution predominantly in the pancreas and liver, with concordant radiotracer uptake in all previously identified lesions; however, lesion conspicuity and tumor-to-background ratios are notably higher compared with [^68^Ga]Ga-DOTA.SA.FAPi. Prolonged blood-pool retention of [^68^Ga]Ga-DOTAGA.(SA.FAPi)_2_ is observed, persisting up to 3 h post-injection. Fused axial PET/CT images demonstrate higher standardized uptake values for [^68^Ga]Ga-DOTAGA.(SA.FAPi)_2_ in normal organs (**b**,**c**) and tumor lesions compared with [^68^Ga]Ga-DOTA.SA.FAPi (**a**) [18].

A clinical study enrolled six cancer patients (male and female, mixed) with progressive disease, who had exhausted all standard treatments. The tumor types included papillary thyroid cancer, breast cancer, and neuroendocrine cancer. The study aimed to evaluate and compare the biodistribution profiles of two radiotracers, [^68^Ga]Ga-DOTA.SA.FAPi and [^68^Ga]Ga-DOTAGA.(SA.FAPi)_2_. No adverse events were reported during or after administration. Both radiotracers exhibited similar physiological uptake patterns in major organs; however, the dimeric compound, [^68^Ga]Ga-DOTAGA.(SA.FAPi)_2_, demonstrated prolonged tumor retention, with sustained uptake up to 3 h post-injection. These findings confirm the authors’ design hypothesis that dimerization of the precursor enhances tumor retention. This study supports a theranostic approach employing the monomeric [^68^Ga]Ga-DOTA.SA.FAPi for diagnostic imaging and the dimeric [^177^Lu]Lu-DOTAGA.(SA.FAPi)_2_ for subsequent targeted radionuclide therapy (Figure 5) [18].

[^177^Lu]Lu-DOTAGA.(SA.FAPi)_2_ exhibits favorable targeting properties, demonstrating considerable potential for therapeutic applications, particularly in treatment-resistant cancers. Initial human studies on its biodistribution and pharmacokinetics indicate that the dimeric design offers key advantages: prolonged tumor retention and a significantly higher tumor-absorbed dose than its monomeric counterpart [19].

The estimated effective half-life (T_e_) of [^177^Lu]Lu-DOTAGA.(SA.FAPi)_2_ was 86.6 h for the left shoulder lesion, 89.5 h for the sternal lesion, and 48.6 h for the right femoral head lesion. Corresponding residence times were 6.4 h, 4.5 h, and 4.0 h, respectively. The calculated absorbed dose estimates were 2.64 Gy/GBq for the left shoulder lesion (lesion mass: 189 g), 89.7 Gy/GBq for the sternum (lesion mass: 3.96 g), and 13.7 Gy/GBq for the right head of the femur (lesion mass: 23.2 g) [19].

**Figure 6:**
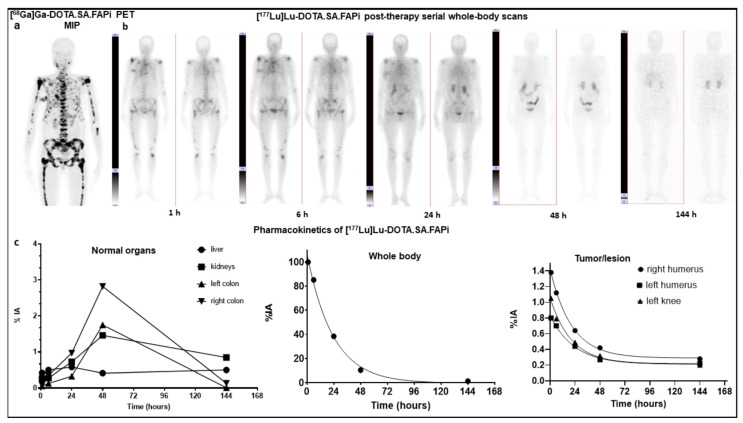
(**a**) Maximum-intensity projection (MIP) image of [^68^Ga]Ga-DOTA.SA.FAPi PET in a 50-year-old woman with follicular variant of papillary thyroid carcinoma, previously treated with radioiodine therapy (cumulative administered activity: 22.2 GBq), demonstrating a soft-tissue density mass in the left shoulder along with multiple skeletal metastases. (**b**) Serial whole-body scintigraphy images acquired at 1 h, 6 h, 24 h, 48 h, and 144 h following administration of 1.48 GBq of [^177^Lu]Lu-DOTAGA.(SA.FAPi)_2_ demonstrate sustained radiotracer retention in metastatic lesions on delayed imaging up to 144 h post-injection. (**c**) Time–activity curves for normal organs (liver, kidneys, colon, pancreas, gall bladder, and salivary glands) derived from regions of interest show peak uptake within 24–48 h followed by a marked decline by 96 h. Whole-body time–activity curve demonstrates gradual systemic clearance over time. Time–activity curves for tumor lesions (left shoulder, sternum, and left head of femur) show sustained radiotracer retention up to 144 h, supporting favorable tumor residence characteristics of [^177^Lu]Lu-DOTAGA.(SA.FAPi)_2_ [19].

While the radioligand accumulates in normal tissues such as the kidneys, liver, and colon, the markedly higher radiation dose delivered to tumors suggests a promising efficacy profile, see Figure 6.

**Figure 7:**
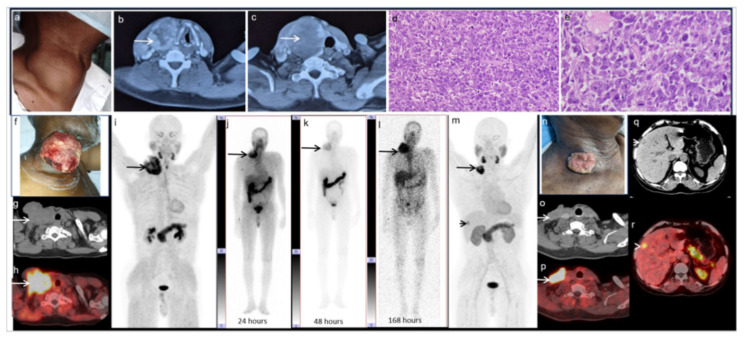
A 56-year-old man with a long-standing anterior neck mass presented with rapid thyroid enlargement over 1 month, associated with dyspea and dysphagia (**a**). Contrast-enhanced CT revealed a large heterogeneously enhancing right thyroid lesion with marked tracheal displacement (**b**,**c**). Highly cellular tumor with pleomorphic polygonal cells, brisk mitotic activity, atypical mitoses, and necrosis (**d**,**e**). He received EBRT (external beam radiotherapy) of 30 Gy in 15 fractions to the neck; however, during EBRT the mass rapidly grew in size at the surgical site (**f**). [^68^Ga]Ga-DOTA.SA.FAPi PET/CT demonstrated intense tracer uptake in the recurrent neck mass on CT (**g**), fused PET/CT (**h**), and MIP images (**i**, arrows). The posttherapy scans (**j**–**l**, arrows) revealed avid and sufficiently prolonged tumor retention, even at 168-h posttreatment (**l**) scan. Follow-up [^68^Ga]Ga-DOTA.SA.FAPi PET/CT demonstrated significant reduction in the recurrent neck mass on MIP (**m**), clinical examination (**n**), CT (**o**), and fused PET/CT (**p**); a new hepatic lesion in segment VIII (**m**,**q**,**r**, arrowheads) [106].

This particular case represents the first patient treated with a FAPI homodimer strategy. A 56-year-old man with a long-standing anterior neck mass presented with rapid thyroid enlargement over 1 month, associated with dyspnea and dysphagia (Figure 7). Following total thyroidectomy with central neck dissection, histopathology demonstrated a highly cellular tumor with pleomorphic polygonal cells, brisk mitotic activity, atypical mitoses, and necrosis. (Immunohistochemistry was negative for thyroid epithelial and neuroendocrine markers, with a MIB-1 labeling index of 45%.) Based on elevated plasma chromogranin-A levels and published grading criteria, a diagnosis of high-grade medullary thyroid carcinoma clinically mimicking anaplastic thyroid cancer was established. Despite external beam radiotherapy (30 Gy/15 fractions), rapid local recurrence developed. The patient received 1.65 GBq of [^177^Lu]Lu-DOTAGA.(SA.FAPi)_2_. Post-therapy imaging showed avid and prolonged tumor retention up to 168 h (j–l, arrows). No grade III/IV toxicity was observed. At 6-week follow-up, the patient showed marked clinical improvement with reduction in pain (VAS 9 → 4), analgesic requirement, dysphagia, tumor size, and plasma chromogranin-A levels (383 → 209 ng/mL). Follow-up [^68^Ga]Ga-DOTA.SA.FAPi PET/CT demonstrated significant reduction in the recurrent neck mass; however, a new hepatic lesion indicated an overall mixed treatment response [106].

The Bal group at AIIMS, India, escalated the administered radiation dose across their research. Beginning with a single administration of 1.48 GBq [19], their latest long-term retrospective study—enrolling 73 pretreated patients—utilized a median activity of 5.5 GBq per cycle over multiple cycles (median: 3; range: 1–9), with follow-up durations ranging from 6 to 40 months. Despite this dose escalation, only 10 patients discontinued treatment due to adverse events, and no instances of immediate toxicity or grade III renal/hepatotoxicity were reported. Compared to lenvatinib, an approved agent for advanced thyroid cancer, [^177^Lu]Lu-DOTAGA.(SA.FAPi)_2_ showed superior tolerability and significantly prolonged median progression-free survival (PFS) (18.3 vs. 29 months). Particularly, among the 73 patients, the 46 with prior tyrosine kinase inhibitor (TKI) exposure showed no statistically significant difference in PFS or overall survival (OS) compared to the TKI-naïve group. These findings position FAP-targeted radioligand therapy as a viable and cost-effective alternative, particularly for patients ineligible for TKI treatment [109].

While homodimeric FAPI derivatives have been used for patient treatment when labelled with ^177^Lu, other trivalent therapeutic isotopes also represent viable candidates. In a long-term study, an innovative tandem beta-alpha therapy strategy was piloted in a subset of eight patients with radioiodine-resistant follicular cell-derived thyroid cancers. They received two cycles of [^177^Lu]Lu-DOTAGA.Glu.FAPi_2_ (median activity: 5.5 GBq) followed by one cycle of [^225^Ac]Ac-DOTAGA Glu.(FAPi)_2_ (median activity: 7.7 MBq) at 8-week intervals. The tandem group demonstrated a trend toward improved OS and PFS, with median survival not reached at analysis; however, the small cohort size limited the statistical significance of these findings [109]. The same tandem therapy regimen was applied in four patients with aggressive medullary thyroid cancer, three of whom demonstrated a treatment response. This strategy is consistent with early PSMA-targeted therapy studies, where low-activity [^225^Ac]Ac-PSMA following [^177^Lu]Lu-PSMA has improved the therapeutic response and the side-effect profiles in progressive patients [114]. As the first clinical application of a ^225^Ac-labeled FAP-dimeric ligand, the DOTAGA.Glu.(FAPi)_2_ platform demonstrates clear translational potential for multi-nuclide therapy; however, the limited pharmacological data from animal models underscore the necessity of complementary preclinical studies. More patients with different types of cancer have been treated meanwhile within that tandem strategy [115,116].

**Figure 8:**
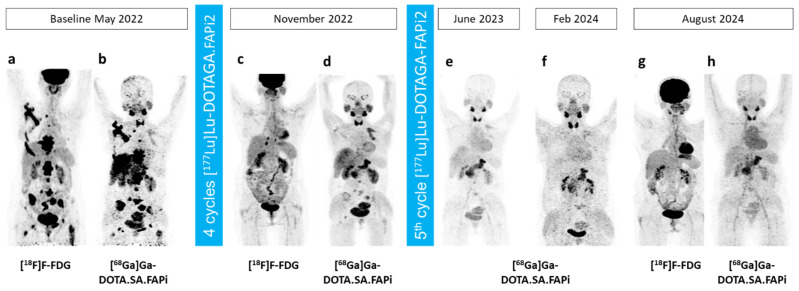
Sequential molecular imaging in a 54-year-old woman with infiltrating ductal carcinoma of the breast (ER-positive, PR-positive, HER2/neu-negative) with liver involvement and extensive skeletal metastases, demonstrating high FDG uptake and marked FAP expression. (**a**,**b**) Baseline imaging acquired in May 2022, showing widespread metastatic disease on [^18^F]F-FDG PET MIP (**a**) with corresponding intense uptake on [^68^Ga]Ga-DOTA.SA.FAPi PET MIP (**b**). Following four cycles of [^177^Lu]Lu-FAPI dimer therapy, interim imaging performed in November 2022 demonstrates interval disease assessment on [^18^F]F-FDG PET (**c**) and [^68^Ga]Ga-DOTA.SA.FAPi PET (**d**). After the fifth cycle of [^177^Lu]Lu-FAPI dimer, follow-up [^68^Ga]Ga-DOTA.SA.FAPi PET images obtained in June 2023 (**e**) and February 2024 (**f**) show sustained disease control with reduced tumor burden. Subsequent imaging in August 2024 includes [^18^F]F-FDG PET (**g**) and [^68^Ga]Ga-DOTA.SA.FAPi PET (**h**), demonstrating continued suppression of metabolically active and FAP-expressing disease. The patient achieved a progression-free duration of 21 months, highlighting durable disease control with FAP-targeted radionuclide therapy [108].

In the treatment study for breast cancer (Figure 8), 19 heavily pretreated patients with advanced breast cancer received [^177^Lu]Lu-DOTAGA.(SA.FAPi)_2_/[^177^Lu]Lu-DOTAGA.Glu.FAPi_2_ with a median administered activity of 5.5 GBq per cycle (range: 2–6 cycles). All patients presented with distant metastases, including bone (19/19), liver (5/19), and brain (7/19). The clinical disease control rate was promising, with 95% of patients achieving disease control, and the objective response rate was 84%. Notably, no severe hematological, renal, or hepatic toxicities, electrolyte imbalances, or grade 3 or 4 adverse events were observed throughout the study. These findings confirm the compound’s favorable safety profile and highlight its potential as a valuable treatment option for patients with limited therapeutic alternatives [108].

The clinical data for the compound [^177^Lu]Lu-DOTAGA.Glu.(FAPi)_2_ indicate its promise as a therapeutic agent for various cancers. In a proof-of-concept study involving a patient with medullary thyroid cancer, the compound showed high tumor uptake and prolonged retention in lesions, alongside significantly reduced uptake in non-target organs like the liver and colon. This profile suggests improved pharmacokinetic and pharmacodynamic properties over the first-generation dimer [^177^Lu]Lu-DOTAGA.(SA.FAPi)_2_ [41].

**Figure 9:**
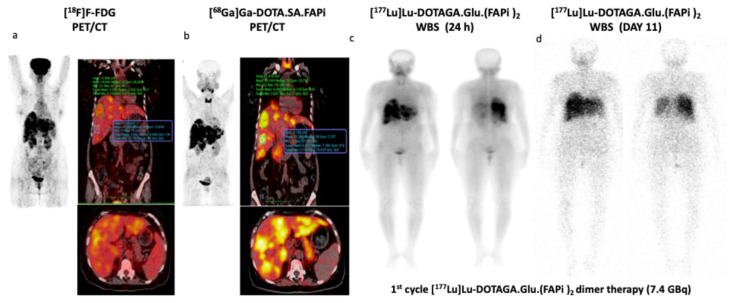
57-year-old woman with undifferentiated pleomorphic liposarcoma originating from the retroperitoneum underwent mesenteric mass resection and received multiple prior systemic therapies, including doxorubicin, ifosfamide, pazopanib, pembrolizumab, and gemcitabine. [^18^F]F-FDG PET/CT (**a**) and [^68^Ga]Ga-DOTA.SA.FAPi PET/CT (**b**). The patient subsequently received two cycles of [^177^Lu]Lu-DOTAGA.Glu.(FAPi)_2_ therapy (cumulative administered activity: 14.8 GBq). Serial post-therapy whole-body scintigraphy following the first cycle demonstrated intense and persistent radiotracer uptake in the hepatic lesion at 24 h (**c**) with sustained retention evident even on delayed imaging at day 11 (**d**) [112].

This potential is further supported by a case report of a 52-year-old female with recurrent glioblastoma. After two cycles of treatment (7.4 GBq per cycle), post-treatment imaging revealed lesion regression and reduced perilesional edema. The patient also showed symptomatic improvement and an enhanced performance status [111]. The efficacy and safety of [^177^Lu]Lu-DOTAGA.Glu.(FAPi)_2_ were evaluated in a small cohort of 10 patients with sarcoma (Figure 9). Tumor uptake was observed for up to 11 days p.i.

The patients received a median cumulative activity of 17.5 GBq (range, 6.3–55.5 GBq) over a median of 3 cycles (range: 1–6 cycles). Follow-up ranged from 4 to 16 months. Treatment was discontinued in one patient due to an adverse event after a single cycle; otherwise, the agent was generally well tolerated. According to PERCIST criteria, a partial response was observed in 33.3% of patients, though no objective responses were noted. The median progression-free survival was approximately 5 months (95% CI, 2.9–7.1 months), and the median overall survival was 8 months (95% CI, 5.5–10.5 months) [112].

A dosimetry study in eight patients treated with up to four cycles (mean activity of 7.1 GBq per cycle) found that while the colon was the critical organ, the absorbed doses remained well below critical thresholds. However, the authors also suggest considering increased administered activities or use of more potent radionuclides such as ^90^Y or alpha emitters [113].

**Figure 10:**
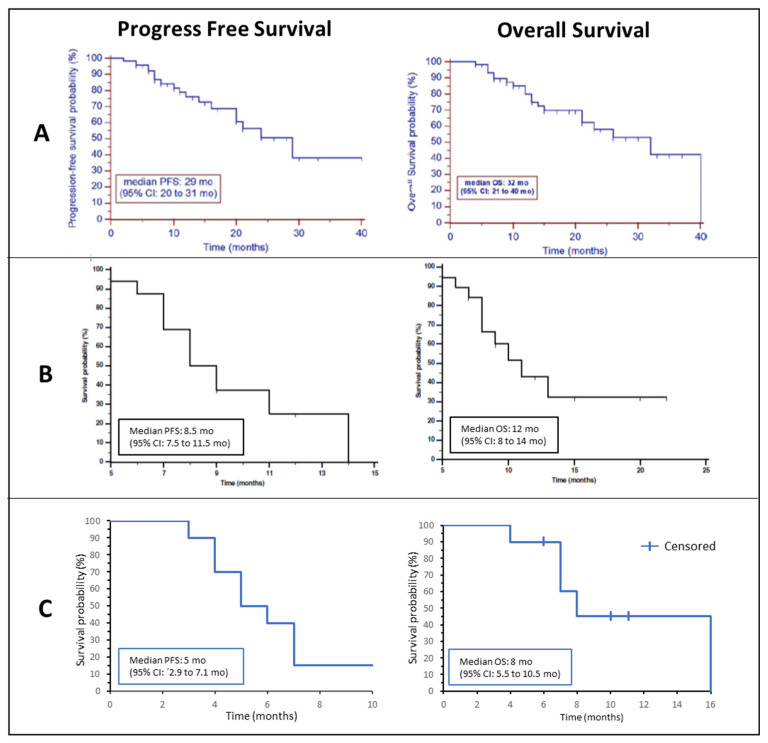
Progress-free survival (PFS) and overall survival (OS) for study participants. (**A**) treatment of [^177^Lu]Lu-DOTAGA.(SA.FAPi)_2_ in patients with radioiodine-resistant follicular cell-derived thyroid cancer [109]; (**B**) treatment of [^177^Lu]Lu-DOTAGA.(SA.FAPi)_2_/[^177^Lu]Lu-DOTAGA.Glu.FAPi_2_ in patients with metastatic breast cancer [108]; (**C**) treatment of [^177^Lu]Lu-DOTAGA.Glu.(FAPi)_2_ in patients with sarcoma [112].

In summary (Figure 10), patients with radioiodine-resistant follicular cell-derived thyroid cancer or metastatic breast cancer demonstrated high therapeutic response rates following treatment with [^177^Lu]Lu-DOTAGA.(SA.FAPi)_2_. Among thyroid cancer patients (N = 73), the median progression-free survival (PFS) was 29 months [95% CI: 14–31 months], and the median overall survival (OS) was 32 months [95% CI: 21–40 months]. No statistically significant differences in PFS or OS were observed between patients who had or had not received prior treatments, including thyroid resection and tyrosine kinase inhibitor (TKI) therapy. In a subgroup of eight patients who received tandem therapy with [^177^Lu]Lu-/[^225^Ac]Ac-DOTAGA.(SA.FAPi)_2_, there was a trend toward improved OS and PFS (not reached vs. 32 months and not reached vs. 29 months, respectively) [109]. In patients with metastatic breast cancer treated with [^177^Lu]Lu-DOTAGA.(SA.FAPi)_2_, median OS was 12 months (95% CI: 8–14 months), and median PFS was 8.5 months (95% CI: 7.5–11.5 months). No severe hematological, renal, or hepatic toxicities were reported [108].

Patients with sarcoma received treatment of [^177^Lu]Lu-DOTAGA.Glu.(FAPi)_2_ demonstrated median PFS of 5 months (95% CI, 2.9–7.1 months) and median OS of 8 months (95% CI, 5.5–10.5 months) [112].

### 8.2. [^68^Ga]Ga-DOTA-2P(FAPI)_2_

Chen et al. group from Xiamen University performed a dosimetry study of [^68^Ga]Ga-DOTA-2P(FAPI)_2_ in three healthy volunteers [43]. Radiotracer uptake was time-independent, stabilizing within 10 min post-injection. The thyroid received the highest effective dose, followed by the liver and lungs. The overall effective dose of [^68^Ga]Ga-DOTA-2P(FAPI)_2_ was comparable to those of [^68^Ga]Ga-FAPI-02 (1.80 × 10^−2^ mSv/MBq) and ^68^Ga-FAPI-04 (1.64 × 10^−2^ mSv/MBq) [12] and higher than that of [^68^Ga]Ga-FAPI-46 (7.80 × 10^−3^ mSv/MBq) [13]. In a head-to-head comparison of diagnostic efficacy in three patients [^68^Ga]Ga-DOTA-2P(FAPI)_2_ demonstrated significantly higher tumor uptake in most lesions than [^68^Ga]Ga-FAPI-46, resulting in clearer visualization of both primary tumors and metastases. Notably, the blood-pool activity of the dimer tracer remained high 4 h post-injection, contrasting with observations in mouse models. Tumor uptake of ^68^Ga-DOTA-2P(FAPI)_2_ showed a slight decrease between 1 and 4 h. No adverse events or changes in vital signs were observed during the 4-h follow-up [43]. Therapeutic applications have not been reported so far.

### 8.3. [^68^Ga]Ga-/[^177^Lu]Lu-LNC1013

A pilot study of seven patients with gastrointestinal cancer reports the first-in-human evaluation of [^68^Ga]Ga-LNC1013. The tracer demonstrated a favorable safety profile and dosimetry. The prostate received the highest average absorbed dose, followed by the liver, lungs, and pancreas. The compound exhibited high and persistent tumor uptake, significantly outperforming [^18^F]F-FDG in detecting primary and metastatic lesions and showing more prolonged retention than [^68^Ga]Ga-FAPI-46. These findings, building on promising preclinical data, establish [^68^Ga]Ga-LNC1013 as a highly promising agent for diagnostic imaging and a strong candidate for future targeted radionuclide therapy [48]. In a clinical comparison of 33 patients with various cancers, [^68^Ga]Ga-LNC1013 demonstrated superior lesion detection in several categories—including primary tumor and liver and peritoneal metastases, achieving higher uptake and image contrast than ^18^F-FDG in selected lesions. Preclinically, [^177^Lu]Lu-LNC1013 exhibited stable and prolonged tumor uptake in mice bearing FAP-positive CT26 tumors, along with superior antitumor efficacy than [^177^Lu]Lu-FAPI-46. The first-in-human study of [^177^Lu]Lu-LNC1013 confirmed a favorable dosimetry profile and excellent safety, with no adverse events reported. Physiological uptake was primarily observed in glandular tissues such as the pancreas, salivary glands, and thyroid, while uptake in non-target organs like the liver, intestines, and kidneys was not significant. Collectively, these findings establish [^68^Ga]Ga-/[^177^Lu]Lu-LNC1013 as a promising theranostic pair for FAP-targeted imaging and radionuclide therapy [76].

## 9. Discussion

The evidence from thyroid, breast, sarcoma, and other cancer studies positions [^177^Lu]Lu-DOTAGA.(SA.FAPi)_2_/DOTAGA.Glu.FAPi_2_ as a highly promising theranostic agent for FAP-expressing cancers. To fully realize its clinical potential, future efforts should focus on refining treatment protocols, establishing long-term outcomes, and optimizing patient selection criteria.

Despite these advances, challenges persist, including variable tumor retention times observed across constructs, incomplete preclinical pharmacology for selected compounds, and the need to better define optimal dosing regimens, radionuclide pairing, and long-term safety. The encouraging early experience with tandem α/β-emission therapy illustrates the potential of multi-nuclide approaches, while ongoing clinical expansion into diverse malignancies underscores broad applicability.

The current amount of clinical evidence establishes the dimeric FAPI radioligands as one of the most promising next-generation theranostic platforms in nuclear oncology. Continued investigation through larger prospective trials, deeper mechanistic studies, and systematic patient stratification will be critical to fully unlock their potential and to define their place in the evolving standard of care for FAP-expressing tumors. Some of the challenging considerations are discussed below.

### 9.1. Equilibrium vs. Kinetics

Multimerization of targeting moieties is well known to medicinal chemistry and pharmacy. Yet, its use in radiopharmaceutical design is relatively new and basically limited to the development of small molecule inhibitors. Next to FAP inhibitors, homodimeric PSMA inhibitors have been investigated to show promising parameters when used as radio-copper-labelled theranostics [117].

Fortunately, in the case of FAP inhibitors, homodimeric constructs clearly help to extend tumor retention times when compared to the same monomeric inhibitor motif. Consistent with this rationale and compared to first-generation FAPI monomers, basically all homodimeric FAP radiotheranostics exhibit prolonged tumor retention and favorable tumor-to-organ ratios at 24 h and later time points post-injection.

Binding affinities of monomeric and dimeric FAP inhibitors have been quantified by various assays in vitro. Accordingly, head-to-head comparisons of the individual molecules are difficult and can be compared only if obtained within the same model. We recommend to include a “reference” FAP inhibitor molecule in all the individual assays for at least relative scaling.

FAP affinities in terms of equilibrium constant K_d_ = k_off_/k_on_ (or K_i_ or IC_50_) do not improve significantly from monomeric to dimeric FAPI analogs, although the DOTAGA.Glu.(FAPi)_2_ represents one of the highest binding affinities measured for almost all FAP inhibitors. Yet it remains in the same order of magnitude (between 1 and 0.1 nanomolar), which rules out affinity aspects for the longer retention time of homodimers compared to monomers of the same inhibitor motif.

The increased tumor uptake and/or tumor residence times that have been reported for FAPI dimers can at least in part be related to their thermodynamic FAP-affinity and their kinetic profile of FAP-binding. For several dimers, significantly higher affinities have been reported than for the corresponding monomeric compounds. A compound’s thermodynamic affinity is represented by the dissociation constant of FAP-binding (K_d_), but in most literature reports, it is proxied by an IC_50_-value or an IC_50_-based K_d_-value. Not all published dimer studies include potency data for reference FAPIs, which complicates the interpretation of published IC_50_-values. Nonetheless, there is a clear trend in literature toward higher affinities for dimers compared to monomers.

Several types of affinity-conferring interactions could underpin these observations. First, the increased overall lipophilicity of dimers and oligomers is an obvious affinity contributor. Compared to a monomeric, radiochelator-derived FAPI, a dimer typically has two relatively lipophilic inhibitor moieties for each highly polar radiochelator moiety. This leads to a higher entropic bonus for dimers upon binding to FAP’s active site. Simultaneous cross-linking of two individual FAP-active sites by the reported FAPI-dimers, however, can be excluded as a factor that contributes to affinity. Although FAP itself occurs as a dimeric protein on the cell surface of CAFs, the two active sites of a FAP dimer are too distant from each other to allow cross-linking. This conclusion has also been drawn in literature by the team of Cazzamalli [23]. Based on molecular docking, all the FAPI oligomers in this paper were deduced to bind to FAP such that only one FAPI unit in each compound is bound in a “canonical” way, i.e., with the carbonitrile in a reversible, covalent engagement with FAP’s nucleophilic serine alcohol. Because the FAPIs in this study have a grossly comparable architecture as other reported dimers and oligomers, it is reasonable to assume that the binding modes presented in this paper are also grossly representative of binding modes of other compounds. Finally, it is possible that additional affinity-conferring interactions are present between FAP and the non-canonically bound part of a dimer. In the absence of high-resolution co-complex structures of FAP and FAPI dimers, however, only speculative claims can be made regarding this possibility. As a cautionary general note, it also deserves mentioning that the competition-based affinity assays that are invariably used in existing dimer literature are unfit for measuring the picomolar compound affinities that have been attributed to some reported molecules. This is because the low picomolar FAPI concentrations that need to be studied in this affinity range are typically comparable to, or even lower than, the concentrations of enzymatically active FAP used in the assays. This does not allow for obtaining consistent and reliable results.

Accordingly, residence time τ = 1/k_off_ became a key direction in designing new FAPI-derivatives, see above. One consideration is that static measurements of binding or just thermodynamic data such as K_d_ are not sufficient, and conventional binding equilibrium assays may not fully identify the kinetics of FAPI–FAP interactions.

Furthermore, even if simultaneous canonical binding of the two FAPI units to FAP is precluded in existing dimers, the divalent structure of these compounds can still significantly influence the two kinetic parameters of binding: the rate of association (k_on_) and the rate of dissociation (k_off_). As mentioned earlier, via the expression K_d_ = k_off_/k_on_, kinetic behavior is also directly linked to thermodynamic affinity. A well-known kinetic feature of di- and multivalent compounds is their relatively high k_on_-rate: because they contain more than one pharmacophore, there is a relatively high probability that at least one of the pharmacophores has the right conformation that is required for binding to the target. Comparably, during compound dissociation from the target, there is also a higher statistical chance that rebinding to the target occurs [28]. This also translates into a net-lower k_off_ rate.

Recent “jump-dilution” assays, in contrast, help to understand the relationships between k_on_ and k_off_ of the binding process. In fact, a recent study confirms the short residence time of monomeric FAPI-derivatives based on the UAMC1110 structure (0.26 h) compared to much longer retention of the homodimer DOTAGA.Glu.(FAPi)_2_ based on the same UAMC1110 structure (6.2 h) [118].

While there is clear evidence of substantially prolonged retention times of FAPI homodimers—both in sophisticated in vitro models, in tumor models, and in patient studies—a final explanation of those observations is still pending. A simultaneous binding of the two FAP inhibitor motifs to two different FAP proteins can be excluded, so eventually there are additional binding phenomena between the homodimeric motif and amino acid sequences of the FAP protein beyond its catalytic triad. A further explanation may lie in the higher fraction of cell internalization and, in particular, slower externalization of the homodimeric compounds. If so, this would represent a kind of “trapping mechanism.” However, cell internalization and externalization phenomena of FAP inhibitors when binding to FAP expressed at CAF sites or tumor cell membranes may differ from the conventional understanding based on e.g., radiolabeled octreotides (attaching the extra-membrane expressed G-protein coupled receptors) or PSMA inhibitors targeting PSMA.

Comprehensive experimental studies on both the thermodynamic and kinetic properties of dimers, however, have so far not been published.

### 9.2. Dimers vs. Multimers

Benefits observed with homodimeric FAPI tracers do not necessarily increase proportionally with higher valency. Instead, compounds with elevated multimeric load, such as DOTA-4P(FAPI)_4_, HexaOncoFAP-DOTAGA, and OctaOncoFAP-DOTAGA, show substantially reduced tumor uptake and inferior FAP binding affinity in vitro and in vivo, indicating an inflection point beyond which multimerization compromises rather than enhances tracer performance. Several non-mutually exclusive mechanisms likely explain the inferior performance observed in high-valency constructs. First, steric crowding and constrained ligand orientation can inhibit optimal FAP engagement [119,120], consistent with the decreased binding affinities measured for hexameric and octameric derivatives. As demonstrated in Figure 3 and Figure 4, the enhanced tumor dosimetry achieved with dimeric FAPI constructs is not accompanied by increased normal-organ exposure, as reflected by stable or improving T/K and T/L ratios over time. Second, increased molecular size elevates hydrodynamic radius, restricts diffusion through the dense tumor extracellular matrix, and impairs stromal penetration [121,122], an especially relevant barrier in fibrotic, CAF-rich malignancies where FAP is predominantly localized. Third, large multimers frequently exceed the renal filtration threshold, shifting clearance toward slower hepatobiliary pathways [123,124], thereby increasing circulation time while reducing tumor-to-background contrast. The clearly low tumor uptake of hexa- and octameric constructs at 24 h p.i. supports these mechanisms. Particularly, even among dimers, pharmacokinetics vary substantially; for example, [^177^Lu]Lu-BiOncoFAP-DOTAGA maintains high tumor retention while [^177^Lu]Lu-LNC1013 demonstrates rapid clearance. Nevertheless, in in vitro and in small animal assays, a tri-homomeric FAP inhibitor was identified along the OncoFAP series to show improved performance compared to homodimer analogs [21]. This highlights that not valency alone, but linker chemistry, spatial arrangement, and physiochemical properties critically manage the in vivo behavior of the derived radioligands.

Finally, the radiometal–chelator pair is not merely a passive labeling tool. While most clinical data have been generated with ^177^Lu-labelled dimers, emerging evidence with ^225^Ac-labeled analogues suggests that switching to alpha emitters may offer complementary advantages, particularly in patients progressing after β-therapy. However, systematic comparisons of the same homodimeric construct labeled with different radiometals (e.g., ^68^Ga vs. ^177^Lu vs. ^225^Ac vs. ^90^Y) are lacking. Such head-to-head evaluations are needed to dissect the contribution of the radiometal–chelator pair to overall pharmacokinetics and therapeutic index.

### 9.3. Cell and Tumor Models

The clinical translation of FAP-targeted dimeric radioligands marks a major milestone in the evolution of precision oncology. By overcoming the limited tumor residence time of first-generation monomers, dimeric FAPI constructs demonstrate substantially enhanced tumor retention, superior tumor-to-background ratios, and compelling therapeutic efficacy across a range of solid malignancies. Among these, [^177^Lu]Lu-DOTAGA.Glu.(FAPi)_2_ currently represents the most clinically advanced compound, supported by robust evidence of survival benefit, high disease control rates, and sustained tolerability even at escalated and repeated dosing. Other homodimers, such as [^177^Lu]Lu-DOTAGA.(SA.FAPi)_2_ and [^177^Lu]Lu-LNC1013, offer distinct pharmacokinetic strengths that support the versatility of FAPI multimerization strategies.

Despite the promising preclinical and, in particular, first clinical proof-of-principle evidence toward potent therapeutic FAPI radiotheranostics, there is a lack of quantitative understanding of the modes of action of existing derivatives. A critical issue is the no-comparability of various preclinical studies due to different assays and models used. The performance of FAPI ligands varies considerably depending on the model employed, reflecting differences in FAP expression density, stromal composition, vascular accessibility, and species-specific tumor biology. A critical challenge is to ensure modes representing tumor microenvironment and CAF, rather than targeting tumor cell membrane-associated FAP.

Importantly, many commonly used xenografted systems express FAP on tumor cells rather than on CAFs, which is inconsistent with human disease, where FAP expression is primarily stromal, spatially heterogeneous, and dynamically regulated. The translational implications of this discrepancy are, for example, highlighted by [^177^Lu]Lu-DOTAGA-2P(FAPI)_2_, which displayed a five-fold lower tumor absorbed dose in PDX models compared to CDX models at 24 h p.i [72]. These findings emphasize that target density alone is insufficient to predict tracer performance, as stromal architecture and ligand accessibility employ equally critical influence. Furthermore, the limited use of immunocompetent, orthotopic, or heterogeneous PDX models restricts evaluation of stromal barriers, tumor perfusion, and intertumoral FAP variability [125,126], thereby reducing the reliability of cross-study comparisons and dose extrapolation. Again, we recommend to include a “reference” FAP inhibitor molecule in all the individual assays for at least relative scaling.

### 9.4. Likely Overestimation of the Therapeutic Efficacy in Tumor Models

FAPI-based radioligands frequently demonstrate exceptionally high tumor uptake and prolonged retention in preclinical models. While these results confirm strong target engagement, they are largely derived from systems with artificially high or homogeneous FAP expression, which likely overestimate clinical efficacy. Extreme FAP overexpression can mask biologically relevant limitations, including insufficient penetration into low-FAP tumor subregions, stromal sink effects that may sequester ligand before tumor saturation, and potential off-target accumulation under conditions where stromal binding capacity is exceeded. Given that human tumors exhibit highly variable and often moderate FAP expression [127], the therapeutic index observed in engineered models should be interpreted as an upper-bound estimate rather than a clinically representative benchmark.

To improve clinical predictability, several priorities needs to be put in place: (1) adoption of standardized model panels capturing low, moderate, and high stromal FAP expression; (2) systematic use of stromal-accurate systems, including CAF-rich PDXs, orthotopic implants, and co-culture or organoid platforms representing heterogeneous tumor microenvironments; (3) integration of clinical FAP expression profiling to calibrate and validate preclinical systems; and (4) inclusion of spatially resolved tumor dosimetry rather than reliance on bulk uptake metrics alone. These steps will be essential for bridging the growing disconnect between encouraging preclinical results and the more unnatural therapeutic responses observed in clinical settings.

### 9.5. Drug-Target Residence Time for Radiotherapeutics

While the definitions of drug-target residence time originate from conventional drug development and therapy, radiotherapeutics present additional specific considerations. First, the amounts of radiotherapeutics are generally very small compared to the administered dose of conventional therapeutics, which has implications for the definition of an EQUILIBRIUM according to L + T = LT. More importantly, however, is the role of the physical half-life of the therapeutic radionuclide: Here, in addition to the kinetics of the dissociation of the LT complex (k_off_), the exponential decay of the radionuclide with its individual physical half-life t½ must be considered.

This could be captured by adopting the well-known definition of the EFFECTIVE HALF-LIFE (t_eff_), which combines the two independent kinetics of the physical half-life and the biological half-life as t_eff_ = (t½ * t_biol_)/(t½ + t_biol_). Applied to the definition of TARGET RESIDENCE TIME, this would result in τ_eff_ = (t½ · τ)/(t½ + τ). The unit of t_eff_ or τ_eff_ remains time, sensibly expressed in hours (h) or days (d).

Example: For a ligand with τ = 48 h at its specific tumor target, the values of τ_eff_ for the derivatives labeled radioactively with ^90^Y (t½ = 64.1 h), ^177^Lu (t½ = 160 h), or ^225^Ac (t½ = 238 h) would be τ_eff_ = 27.4 h, 36.9 h, or 39.9 h. The shorter the ligand’s tumor residence time, the shorter the effective residence time becomes: for τ = 24 h, τ_eff_ gives 17.5 h, 20.9 h, and 21.8 h, respectively; for τ = 6 h, τ_eff_ results in 5.49 h, 5.78 h, and 5.85 h, respectively. In other words, the further the ligand’s residence time falls below the half-life of the radioisotope, the shorter the effective residence time becomes, and the more the values of the effective residence time converge for radioisotopes with different physical half-lives.

Third, for radiotherapeutics, it must be borne in mind that the values of the effective tumor residence time are a direct measure of the radiation dose therapeutically effective at the tumor. The inevitable conclusion is that medicinal chemistry must work intensively to optimize the residence time at the tumor IN RELATION TO THE PHYSICAL HALF-LIFE of the radioisotope. From the authors’ perspective, values of τ on the order of half the physical half-life t½ would be appropriate: this already delivers two-thirds of the dose that would be calculated for a ratio of τ = t½. Tumor residence times of the FAPI homodimers in the range of 3–5 days, as already achieved with the current derivatives, thus provide very good conditions for systematic clinical trials.

In addition to the desired prolonged retention time of the radiotherapeutic agent at its specific tumor target, a time-dependent increase in the ratio between the local concentration of the radiotherapeutic agent at the target and its concentrations in the circulation, excretory organs, or healthy organs occurs—provided that the binding between the radiotherapeutic agent and the target is highly selective. Regarding the accumulation ratios between tumor and blood, tumor and kidney, tumor and liver, etc., one would logically expect these values to increase substantially over time. Preclinical and clinical data to date on FAPI homodimers confirm this impressively. Interestingly, this may represent a characteristic advantage of the concept of homodimerization of FAP inhibitors over other approaches to prolonging tumor retention time, particularly with regard to albumin-binding FAPI monomers.

### 9.6. Toward Patient Selection for Homomultimeric FAPI Radiotheranostics

While definitive inclusion and exclusion criteria must await prospective clinical trials, the evidence compiled in this review permits several evidence-based observations to guide initial patient selection. A formal, validated clinical algorithm for patient selection in homomultimeric FAPI radiotheranostics is not yet available; however, based on the evidence compiled in this review, an evidence-informed framework—encompassing FAP expression confirmation, tumor type assessment, organ-specific safety evaluation, bone marrow reserve, and dosing regimen—can serve as a foundation for future algorithm development.

**FAP expression level.** High FAP expression consistently correlates with superior therapeutic outcomes. As summarised in Table 5, in high-FAP-expressing models such as SK-RC-52.hFAP, dimeric and trimeric constructs achieved complete remissions, whereas efficacy was markedly diminished in low-FAP models such as CT-26.hFAP. Given that most preclinical data derive from FAP-overexpressing CDX models—a limitation discussed in the subsection “9.4. Likely overestimation of the therapeutic efficacy in tumour models”—clinical selection should employ quantitative FAP PET/CT (e.g., using SUVmax thresholds) or immunohistochemistry to identify patients most likely to benefit.

**Tumor types most likely to benefit.** The strongest clinical evidence to date supports homomultimeric FAP-targeted therapy in radioiodine-refractory follicular cell-derived thyroid cancer and medullary thyroid cancer (73 patients, median PFS 29 months, Table 7 and Ballal et al. 2025 [109]), metastatic breast cancer (objective response rate 84%, Yadav et al. 2023 [108]), and sarcomas (a subset achieving partial responses, Ballal et al. 2025 [112]). Other stroma-rich malignancies—including pancreatic cancer, glioblastoma, and hepatocellular carcinoma—remain promising candidates based on biological rationale and emerging preliminary data.

**Potential exclusion criteria.** Based on current evidence, exclusion should be considered for (i) tumors demonstrating low or absent FAP uptake on [^68^Ga]Ga-FAPI PET/CT; (ii) patients with critical organ involvement where physiological uptake (e.g., thyroid, liver for certain dimeric constructs, as reported by Zhao et al. 2021 [43] would result in unacceptable radiation exposure; and (iii) individuals with poor baseline bone marrow reserve, notwithstanding the absence of grade ≥ 3 haematological toxicity in published series.

Prospective trials utilising standardised FAP PET quantification and stroma-accurate preclinical models—particularly patient-derived xenografts (PDX) that better recapitulate human tumour microenvironment heterogeneity—are urgently needed to transform these evidence-informed trends into validated clinical selection criteria.

## 10. Conclusions and Outlook

Despite the challenges in in vitro and preclinical models in vivo, or, generally speaking, in fully understanding the rationale of FAPI homodimerization, the prolonged tumor residence by using FAPI homodimers in patients while keeping tracer accumulation in healthy organs at very low levels represents a dramatic progress in treating various kinds of cancer in a pan-tumor approach.

As summarized in Table 7, individual FAPI homodimers have been applied for patient therapy. The majority applies to DOTAGA.(SA.FAPi)_2_ and DOTAGA.Glu.(FAPi)_2_, covering more than 100 patient cases. Several types of cancers have been treated with clear effects on overall and progression-free survival. In particular, the quality of life improved for many patients beyond overall and progression-free survival. Because basically all those published therapeutic studies represent a “real-life-scenario” of patient treatment, many important challenges remain to be addressed prior to the final design of formal theranostic studies.

***Impact of the preclinical model variability***: A major translational limitation identified in the current literature is the lack of standardized, biologically representative models, in particular related to (i) in vitro binding assays (both in affinity and kinetics of binding), (ii) cell internalization and externalization, and (iii) tumor models. Once again, we recommend including a “reference” radiolabeled FAP inhibitor molecule in all the individual assays to achieve better comparability among the many published data.

***Type of cancer***: Although there is a principal perception that FAPI theranostics are of pan-cancer profile, there seems to be a need to identify the most important types of cancer to be addressed first—based on different criteria, e.g., unmet clinical need.

***Injected activities***: Injected activities were chosen “traditionally” to amount to 7.4 GBq. Thanks to relatively low dose of [^177^Lu]Lu-DOTAGA.Glu.(FAPi)_2_ to healthy organs, injected activities of up to 11 GBq are being well tolerated [113].

***Isotopes***: Among the therapeutic radionuclides, ^90^Y, ^177^Lu, and ^225^Ac have been applied, with ^161^Tb obviously waiting for its time to come. Each offers a certain rationale. ^90^Y may offer beneficial crossfire effects for high-energetic, long-ranging beta-electrons of ^90^Y, eventually covering both tumor stroma and central cancer tissue. ^177^Lu, nevertheless, works well with its low-range beta-electrons. The alpha-emitter ^225^Ac is an interesting option. Its action would focus on CAF (or FAP expressed on tumor cell membranes) “only”: there is already first clinical evidence that ^225^Ac-DOTAGA.Glu.(FAPi)_2_ represents a useful extension relative to ^177^Lu-DOTAGA.Glu.(FAPi)_2_. More therapeutic isotopes are under consideration, such as ^212^Pb, ^211^At, and ^131^I, each of them needing modifications in the linker part and labeling moiety of the FAPi homodimer design.

**Figure 11:**
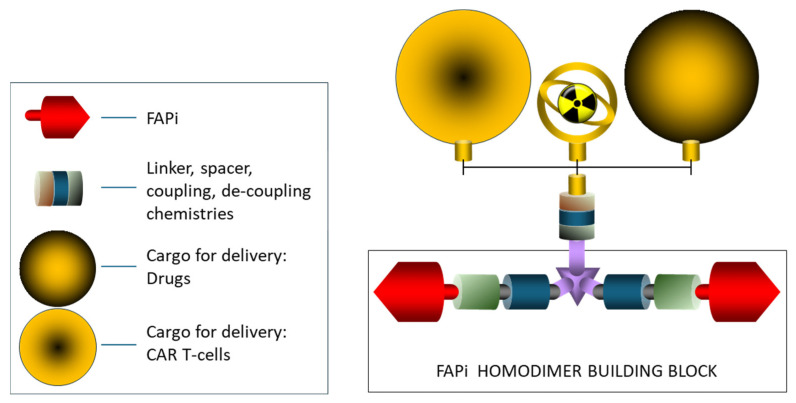
Simplified illustration of the trislinker-FAPI homodimer platform. In addition to systematic variations in linker, spacer, and coupling chemistry, the radiolabeling moiety is flexible. Moreover, instead of the radiolabel for FAPI homodimer building block could deliver cargos for different purposes.

***Platform philosophy***: Current data support dimeric FAPI constructs as the most pharmacokinetically balanced platform for FAP-based theranostics. Rational optimization of multimer design, paired with biologically faithful and standardized modeling frameworks, will be central to advancing FAPI radiotheranostics toward reliable clinical impact. Modifying various constituents of the current trislinker-FAPI homodimer platform (see Figure 11), such as linker and/or spacer and/or chelator and/or isotope or even the inhibitor motif itself, may further improve the ADME profile of this class of radiotherapeutics. There is recent evidence that increasing distance between the tris-linker center and the inhibitor or the chelator may yield further prolongation of tumor residence time and pharmacology [42]. In parallel, there is ongoing research and development toward using the FAPI homodimer as a building block for different applications, e.g., the delivery of CAR T-cell constructs [128].

In summary, homomultimerization, particularly homodimerization, of FAP inhibitors represents a validated and clinically translatable strategy to overcome the short tumor retention times that limit first-generation FAPI monomers for radionuclide therapy. Preclinical and clinical evidence consistently demonstrate that optimized dimeric constructs, such as [^177^Lu]Lu-DOTAGA.(SA.FAPi)_2_ and [^177^Lu]Lu-DOTAGA.Glu.(FAPi)_2_, achieve significantly prolonged tumor residence, favorable tumor-to-background ratios, and encouraging therapeutic responses across multiple solid tumor types. However, increasing valency beyond dimers does not proportionally enhance performance; higher-order multimers (tetramers, hexamers, and octamers) often suffer from steric hindrance, reduced binding affinity, and compromised tumor uptake. A critical barrier to cross-study comparison remains the high variability in preclinical models, cell lines, and assay conditions. Therefore, including a standardized reference FAP inhibitor across all in vitro and in vivo evaluations is essential for meaningful data integration. Prospective head-to-head clinical trials directly comparing dimeric versus monomeric FAPI radioligands are needed to definitively establish whether the improved preclinical tumor retention translates into superior clinical outcomes. Overall, dimeric FAPI radiotheranostics offer a robust and versatile platform for personalized cancer treatment, with ongoing opportunities to further optimize linker design, radionuclide pairing, and combination therapy regimens.

## 11. Patents

SCV holds the following active patents related to dimeric FAPI radioligands: CN114796533B, JP7653082B2, KR102910082B1, AU2019365377B2, and US11833229B2.

## Figures and Tables

**Table 6:** Summary of dose escalation studies.

Compound	Animal Model	Precursor Mass	Radioactivity	Optimal Dose
[^177^Lu]Lu-DOTAGA.(SA.FAPi)_2_ [103]	wild-type 4T1 CDX	10, 50 nmol/kg	18.5 MBq	10 nmol/kg
[^177^Lu]Lu-BiOncoFAP [104]	SK-RC-52.hFAP CDX	3–2250 nmol/kg	n.a.	90–250 nmol/kg
[^177^Lu]Lu-OncoFAP-23 [104]	SK-RC-52.hFAP CDX	3–2250 nmol/kg	n.a.	90–250 nmol/kg
[^68^Ga]Ga-DO3A.Glu.(FAPi)_2_ [105]	PC3-CDX	10–1500 pmol/mouse	0.3 MBq/100 pmol (0.03–4 MBq)	350–600 pmol (* 16.7–28.6 nmol/kg)
[^68^Ga]Ga-DOTAGA.Glu.(FAPi)_2_ [105]	PC3-CDX	10–1500 pmol/mouse	0.3 MBq/100 pmol (0.03–4 MBq)	350–600 pmol (* 16.7–28.6 nmol/kg)
[^68^Ga]Ga-DOTAGA.(SA.FAPi)_2_ [105]	PC3-CDX	10–1500 pmol/mouse	0.3 MBq/100 pmol (0.03–4 MBq)	350–600 pmol (* 16.7–28.6 nmol/kg)

* The weight of scarified mice was between 20 and 22 g.

**Table 7:** Summary of clinical application of different FAPI conjugated radiopharmaceuticals.

Compound	Application	Healthy Volunteers	Disease	Number of Patients	Dosimetry Study	Dosage (/Cycle)	Ref.
[^68^Ga]Ga-DOTAGA.(SA.FAPi)_2_	imaging	no	papillary thyroid cancer, breast cancer, neuroendocrine cancer	6	no	48.1–88.8 MBq (Mean = 74)	[18]
[^177^Lu]Lu-DOTAGA.(SA.FAPi)_2_	therapy	no	breast cancer, thyroid cancer, paraganglioma	7	yes	1.48 GBq	[19]
			medullary thyroid cancer	1	no	1.65 GBq	[106]
			thyroid cancer	15	yes	Median = 2.6 GBq	[107]
			breast cancer	19	no	Median = 5.5 GBq	[108]
			follicular cell-derived thyroid cancers *	73	no	Median = 5.5 GBq	[109]
[^225^Ac]Ac-DOTAGA.Glu.(FAPi)_2_	therapy	no	follicular cell-derived thyroid cancers	8	no	Median = 7.7 MBq	[109]
			medullary thyroid cancer	4	no	Median = 6.66 MBq	[110]
[^177^Lu]Lu-DOTAGA.Glu.(FAPi)_2_	therapy	no	medullary thyroid cancer	1	no	5.5 GBq	[41]
			follicular cell-derived thyroid cancers *	73	no	Median = 5.5 GBq	[109]
			medullary thyroid cancer	19	no	Median = 5.5 GBq	[110]
			glioblastoma	1	no	7.4 GBq	[111]
			sarcoma	10	no	Median = 6.0 GBq	[112]
			5 different cancer types **	8	yes	Mean = 7.1 ± 0.5 GBq	[113]
[^68^Ga]Ga-DOTA-2P(FAPI)_2_	imaging	yes	nasopharyngeal carcinoma, papillary thyroid carcinoma, hepatocellular carcinoma	3	yes	no mention	[43]
[^68^Ga]Ga-LNC1013	imaging	no	gastrointestinal cancer	7	yes	219.8–256.4 MBq	[48]
	Imaging	no	12 different cancer types ***	33	no	148 ± 52 MBq	[76]
[^177^Lu]Lu-LNC1013	therapy	No	metastatic gastric cancer	3	yes	1.86–2.04 GBq	[76]

* In the initial phase, patients received [^177^Lu]Lu-DOTAGA.(SA.FAPi)_2_ for 1–2 cycles. The patients were later treated with [^177^Lu]Lu-DOTAGA.Glu.(FAPi)_2_ after standardization. ** advanced prostate carcinoma, papillary thyroid carcinoma, sarcoma, pancreatic carcinoma, pleural mesothelioma. *** colorectal cancer, gastric cancer, lung cancer, cervical cancer, pancreatic cancer, esophageal cancer, ureteral carcinoma, rhabdomyosarcoma, lymphoma, ovarian cancer, primary unknown.

## Data Availability

The original contributions presented in this study are included in the article/Supplementary Material. Further inquiries can be directed to the corresponding authors.

## Supplementary Materials

The following supporting information can be downloaded at: https://www.mdpi.com/article/10.3390/molecules31122124/s1, Table S1: Tumor-to-liver ratios after administration of different FAPI conjugated radiopharmaceuticals in animal models at 1, 2, 4, 8, 17, 24, 48, 72, and 96 h post-injection. Table S2: Tumor-to-kidney ratios after administration of different FAPI-conjugated radiopharmaceuticals in animal models at 1, 2, 4, 8, 17, 24, 48, 72, and 96 h post-injection. Table S3: Tumor-to-blood ratios after administration of different FAPI-conjugated radiopharmaceuticals in animal models at 1, 2, 4, 8, 17, 24, 48, 72, and 96 h post-injection. Table S4: Tumor-to-heart ratios after administration of different FAPI-conjugated radiopharmaceuticals in animal models at 1, 2, 4, 8, 17, 24, 48, 72, and 96 h post-injection.
