# Supplementary material for: Homomultimeric FAP Inhibitor-Based Radioligands for Cancer Theranostics: Design Principles, Structure–Function Relationships, and Preclinical Performance
Source: Molecules. 2026 Jun 16;31(12):2124. doi: 10.3390/molecules31122124 (PMC13304966; doi:10.3390/molecules31122124)
Supplement: Supplementary file 1 [file molecules-31-02124-s001.zip › molecules-4303149-supplementary.pdf]

Supplementary Materials

Homomultimeric FAP Inhibitor-Based Radioligands for Cancer Theranostics: Design Principles, Structure–Function Relationships, and Preclinical Performance

Zhiyang Wu [1,*], Eleni Gourni [2], Sanjana Ballal [3], Pieter Van der Veken [4] and Frank Roesch [5,*]

[1] SCV Spezial-Chemikalien-Vertrieb GmbH, 10243 Berlin, Germany
[2] Department of Nuclear Medicine, Inselspital, Bern University Hospital, CH-3010 Bern, Switzerland; eleni.gourni@insel.ch
[3] Department of Nuclear Medicine, All India Institute of Medical Sciences, New Delhi 110029, India; mail.sanjanaballal87@gmail.com
[4] Laboratory of Medicinal Chemistry, Department of Pharmaceutical Sciences, University of Antwerp, 2610 Wilrijk, Belgium; pieter.vanderveken@uantwerpen.be
[5] Department of Chemistry–TRIGA Site, Johannes Gutenberg-University Mainz, 55128 Mainz, Germany
* Correspondence: zhiyang.wu@scv-company.com (Z.W.); frank.roesch@uni-mainz.de (F.R.)

Table S1. Tumor-to-liver ratios after administration of different FAPI conjugated radiopharmaceuticals in animal models at 1, 2, 4, 8, 17, 24, 48, 72, and 96h post injection.

	Precursor	Animal Model	Tumor/liver								
			1h	2h	4h	8h	17h	24h	48h	72h	96h
Dimeric	^{68}Ga-DOTAGA.(SA.FAPi)$_2$ [1]	PC3 CDX	0.9	1.3	-	-	-	-	-	-	-
	^{68}Ga-DOTAGA.(SA.FAPi)$_2$ [1]	U87MG CDX	1.6	1.4	-	-	-	-	-	-	-
	^{177}Lu-DOTAGA.(SA.FAPi)$_2$ [1]	PC3 CDX	-	-	1.2	-		0.5	0.5	0.3	0.3
	*^{68}Ga-DOTAGA.Glu.(FAPi)$_2$[2]	PC3 CDX	5.3	11.7		-	-	-	-	-	-
	*^{177}Lu-DOTAGA.Glu.(FAPi)$_2$[2]	PC3 CDX	-	-	8.0	-	-	2.8	2.8	2.3	1.7
	^{68}Ga-DO3A.Glu.(FAPI)$_2$[3]	PC3 CDX	4.8	6.2	-	-	-	-	-	-	-
	^{177}Lu-DO3A.Glu.(FAPI)$_2$[3]	PC3 CDX	-	-	9.0	-	-	4.4	2.9	1.9	1.4
	*^{177}Lu-BiOncoFAP-DOTAGA [4]	HT-1080.hFAP CDX	20	-	34	-	34	34	-	-	-
	^{177}Lu-BiOncoFAP-11 [5]	HT-1080.hFAP CDX	-	-	-	-	-	14.5	12.0	16.0	-
	^{68}Ga-DOTA-2P(FAPI)$_2$ [6]	HCC-PDX	4.0	-	-	-	-	-	-	-	-
	*^{177}Lu-DOTA-2P(FAPI)$_2$ [7]	HCC-PDX Model	2.0	-	1.6	-	-	1.3	1.0	-	-
	*^{177}Lu-DOTA-2P(FAPI)$_2$ [7]	HT-1080.hFAP CDX	10.5	-	11.0	-	-	12.0	10.0	-	-
	^{99m}Tc-TPPTS-HF$_2$ [8]	HT-1080.hFAP CDX		-	5.9	-	-	-	-	-	-
	^{99m}Tc-TPPTS-HF$_2$ [8]	U87MG CDX	5.0	6.5	4.2	-	-	-	-	-	-
	^{99m}Tc-L$_2$-TPPTS[9]	U87MG CDX	7.5	-	3.9	-	-	-	-	-	-
	^{99m}Tc-L$_2$-PDA[9]	U87MG CDX	3.8	-	3.5	-	-	-	-	-	-
	*^{68}Ga-LNC-1013 [10]	HT-1080.hFAP CDX	4.7	3.0	-	-	-	-	-	-	-
	^{177}Lu LNC-1013[11]	CT26-FAP CDX	-	-	1.6	-	-	1.0	0.9	0.5	0.5
	*^{68}Ga-FAPI-3 [10]	HT-1080.hFAP CDX	2.6	2.8	2.0	-	-	-	-	-	-
	*^{68}Ga-FAPI-5 [10]	HT-1080.hFAP CDX	6.5	4.2	-	-	-	-	-	-	-
	^{68}Ga-HBED-CC-FAPI-02 dimer [12]	U87MG CDX	5.7	5.8	-	-	-	-	-	-	-
	*^{18}F-ND-bisFAPI [13]	A549-FAP CDX	20.0	30.8	51.7	-	-	-	-	-	-
	^{177}Lu-ND-bisFAPI [13]	A549-FAP CDX	1.9	-	2.2	-	-	4.1	-	0.9	-
	^{177}Lu-FAPI-46-F1D [14]	HT-1080.hFAP CDX	-	-	15.6	-	-	4.7	-	2.9	-
	^{177}Lu-FAPI-46-F1D [14]	HEK-293.hFAP CDX	-	-	20.7	-	-	4.4	-	0.9	-
Trimeric	^{177}Lu-OncoFAP-23 [15]	SK-RC-52.hFAP CDX	-	-	-	-	-	61.5	61.9	1294.6	41.0
Tetrameric	*^{177}Lu-TetraOncoFAP-DOTAGA [16]	SK-RC-52.hFAP CDX	-	-	-	-	-	11.4	-	-	2.4
	^{177}Lu-DOTA-4P(FAPI)$_4$ [17]	HT-1080.hFAP CDX	-	-	-	-	-	3.3	2.7	2.9	2.0
Hexametric	*^{177}Lu-HexaOncoFAP-DOTAGA [16]	SK-RC-52.hFAP CDX	-	-	-	-	-	0.032	-	-	0.02
Octameric	*^{177}Lu-OctaOncoFAP-DOTAGA [16]	SK-RC-52.hFAP CDX	-	-	-	-	-	0.02	-	-	0.1

* the values from references were read and calculated from the graphs and therefore do not indicate precise values.

Table S2. Tumor-to-kidney ratios after administration of different FAPI conjugated radiopharmaceuticals in animal models at 1, 2, 4, 8, 17, 24, 48, 72, and 96h post injection.

	Precursor	Animal Model	Tumor/kidney								
			1h	2h	4h	8h	17h	24h	48h	72h	96h
Dimeric	^{68}Ga-DOTAGA.(SA.FAPi)$_2$ [1]	PC3 CDX	0.8	1.4	-	-	-	-	-	-	-
	^{68}Ga-DOTAGA.(SA.FAPi)$_2$ [1]	U87MG CDX	1.0	1.3	-	-	-	-	-	-	-
	^{177}Lu-DOTAGA.(SA.FAPi)$_2$ [1]	PC3 CDX	-	-	1.3	-	-	1.0	1.1	0.9	1.3
	*^{68}Ga-DOTAGA.Glu.(FAPi)$_2$[2]	PC3 CDX	2.0	3.5	-	-	-	-	-	-	-
	*^{177}Lu-DOTAGA.Glu.(FAPi)$_2$[2]	PC3 CDX	-	-	6.4	-	-	4.7	5.5	7.0	12.5
	^{68}Ga-DO3A.Glu.(FAPI)$_2$[3]	PC3 CDX	2.9	4.6	-	-	-	-	-	-	-
	^{177}Lu-DO3A.Glu.(FAPI)$_2$[3]	PC3 CDX	-	-	10.3	-	-	7.9	7.6	8.2	7.9
	*^{177}Lu-BiOncoFAP-DOTAGA [4]	HT-1080.hFAP CDX	7	-	10	-	10	12	-	-	-
	^{177}Lu-BiOncoFAP-11 [5]	HT-1080.hFAP CDX	-	-	-	-	-	4	4	6	-
	^{68}Ga-DOTA-2P(FAPI)$_2$ [6]	HCC-PDX	1.6	-	1.3	-	-	-	-	-	-
	*^{177}Lu-DOTA-2P(FAPI)$_2$ [7]	HCC-PDX Model	2.0	-	3.0	-	-	2.0	2.0	-	-
	*^{177}Lu-DOTA-2P(FAPI)$_2$ [7]	HT-1080.hFAP CDX	7.0	-	7.5	-	-	11.0	10.0	-	-
	^{99m}Tc-TPPTS-HF$_2$ [8]	HT-1080.hFAP CDX	-	-	8.4	-	-	-	-	-	-
	^{99m}Tc-TPPTS-HF$_2$ [8]	U87MG CDX	3.2	3.3	1.8	-	-	-	-	-	-
	^{99m}Tc-L$_2$-TPPTS[9]	U87MG CDX	2.9	-	3.3	-	-	-	-	-	-
	^{99m}Tc-L$_2$-PDA[9]	U87MG CDX	4.8	-	3.6	-	-	-	-	-	-
	*^{68}Ga-LNC-1013 [10]	HT-1080.hFAP CDX	3.2	4.8	18.6	-	-	-	-	-	-
	^{177}Lu LNC-1013[11]	CT26-FAP CDX	-	-	2.0	-	-	2.0	1.8	1.7	2.0
	*^{68}Ga-FAPI-3 [10]	HT-1080.hFAP CDX	2.9	4.4	2.5	-	-	-	-	-	-
	*^{68}Ga-FAPI-5 [10]	HT-1080.hFAP CDX	2.6	2.7	6.0	-	-	-	-	-	-
	^{68}Ga-HBED-CC-FAPI-02 dimer [12]	U87MG CDX	15.1	9.3	-	-	-	-	-	-	-
	*^{18}F-ND-bisFAPI [13]	A549-FAP CDX	7.3	7.3	7.8	-	-	-	-	-	-
	^{177}Lu-ND-bisFAPI [13]	A549-FAP CDX	1.8	-	2.0	-	-	6.5	-	1.9	-
	^{177}Lu-FAPI-46-F1D [14]	HT-1080.hFAP CDX	-	-	8.4	-	-	6.3	-	4.1	-
	^{177}Lu-FAPI-46-F1D [14]	HEK-293.hFAP CDX	-	-	13.4	-	-	6.3	-	2.0	-
Trimeric	^{177}Lu-OncoFAP-23 [15]	SK-RC-52.hFAP CDX	-	-	-	-	-	36.3	45.6	24.8	22.0
Tetrameric	*^{177}Lu-TetraOncoFAP-DOTAGA [16]	SK-RC-52.hFAP CDX	-	-	-	-	-	38.0	-	-	2.8
	^{177}Lu-DOTA-4P(FAPI)$_4$ [17]	HT-1080.hFAP CDX	-	-	-	-	-	3.6	3.0	3.1	2.4
Hexametric	*^{177}Lu-HexaOncoFAP-DOTAGA [16]	SK-RC-52.hFAP CDX	-	-	-	-	-	2.5	-	-	0.8
Octameric	*^{177}Lu-OctaOncoFAP-DOTAGA [16]	SK-RC-52.hFAP CDX	-	-	-	-	-	2.0	-	-	1.5

* the values from references were read and calculated from the graphs and therefore do not indicate precise values.

Table S2. Tumor-to-blood ratios after administration of different FAPI conjugated radiopharmaceuticals in animal models at 1, 2, 4, 8, 17, 24, 48, 72, and 96h post injection.

	Precursor	Animal Model	Tumor/blood								
			**1h**	**2h**	**4h**	**8h**	**17h**	**24h**	**48h**	**72h**	**96h**
Dimeric	^{68}Ga-DOTAGA.(SA.FAPi)$_2$ [1]	PC3 CDX	0.3	0.5	-	-	-	-	-	-	-
	^{68}Ga-DOTAGA.(SA.FAPi)$_2$ [1]	U87MG CDX	0.4	0.5	-	-	-	-	-	-	-
	^{177}Lu-DOTAGA.(SA.FAPi)$_2$ [1]	PC3 CDX	-	-	0.7	-	-	1.5	3.8	8.2	18.0
	*^{68}Ga-DOTAGA.Glu.(FAPi)$_2$[2]	PC3 CDX	5.3	7.0	-	-	-		-	-	-
	*^{177}Lu-DOTAGA.Glu.(FAPi)$_2$[2]	PC3 CDX	-	-	2.3	-	-	7.0	-	-	-
	^{68}Ga-DO3A.Glu.(FAPI)$_2$[3]	PC3 CDX	9.7	8.9	-	-	-	-	-	-	-
	^{177}Lu-DO3A.Glu.(FAPI)$_2$[3]	PC3 CDX	-	-	6.6	-	-	8.2	13.3	24.4	51.5
	*^{177}Lu-BiOncoFAP-DOTAGA [4]	HT-1080.hFAP CDX	30	-	65	-	110	150	-	-	-
	^{177}Lu-BiOncoFAP-11 [5]	HT-1080.hFAP CDX	-	-	-	-	-	652.0	2408	3628	-
	^{68}Ga-DOTA-2P(FAPI)$_2$ [6]	HCC-PDX	-	-	-	-	-	-	-	-	-
	*^{177}Lu-DOTA-2P(FAPI)$_2$ [7]	HCC-PDX Model	0.9		1.2	-	-	8.0	-	-	-
	*^{177}Lu-DOTA-2P(FAPI)$_2$ [7]	HT-1080.hFAP CDX	5.3		6.0	-	-	-	-	-	-
	^{99m}Tc-TPPTS-HF$_2$ [8]	HT-1080.hFAP CDX	-	-	-	-	-	-	-	-	-
	^{99m}Tc-TPPTS-HF$_2$ [8]	U87MG CDX	1.4	2.5	2.1	-	-	-	-	-	-
	^{99m}Tc-L$_2$-TPPTS[9]	U87MG CDX	3.0	-	3.2	-	-	-	-	-	-
	^{99m}Tc-L$_2$-PDA[9]	U87MG CDX	3.5	-	3.0	-	-	-	-	-	-
	*^{68}Ga-LNC-1013 [10]	HT-1080.hFAP CDX	2.4	3.0	-	-	-	-	-	-	-
	^{177}Lu LNC-1013[11]	CT26-FAP CDX	-	-	1.3	-	-	5.9	15.2	16.5	18.0
	*^{68}Ga-FAPI-3 [10]	HT-1080.hFAP CDX	2.7	2.8	3.0	-	-	-	-	-	-
	*^{68}Ga-FAPI-5 [10]	HT-1080.hFAP CDX	4.3	4.0	6.0	-	-	-	-	-	-
	^{68}Ga-HBED-CC-FAPI-02 dimer [12]	U87MG CDX	5.9	3.8		-	-	-	-	-	-
	*^{18}F-ND-bisFAPI [13]	A549-FAP CDX	-	-	-	-	-	-	-	-	-
	^{177}Lu-ND-bisFAPI [13]	A549-FAP CDX	1.3	-	2.1	-	-	29.6	-	74.0	-
	^{177}Lu-FAPI-46-F1D [14]	HT-1080.hFAP CDX	-	-	8.7	-	-	10.7	-	89.3	-
	^{177}Lu-FAPI-46-F1D [14]	HEK-293.hFAP CDX	-	-	11.4	-	-	10.0	-	28.6	-
Trimeric	^{177}Lu-OncoFAP-23 [15]	SK-RC-52.hFAP CDX	-	-	-	-	-	228.6	588.7	7913.2	3504.0
Tetrameric	*^{177}Lu-TetraOncoFAP-DOTAGA [16]	SK-RC-52.hFAP CDX	-	-	-	-	-	-	-	-	-
	^{177}Lu-DOTA-4P(FAPI)$_4$ [17]	HT-1080.hFAP CDX	-	-	-	-	-	11.9	-	-	-
Hexametric	*^{177}Lu-HexaOncoFAP-DOTAGA [16]	SK-RC-52.hFAP CDX	-	-	-	-	-	-	-	-	-
Octameric	*^{177}Lu-OctaOncoFAP-DOTAGA [16]	SK-RC-52.hFAP CDX	-	-	-	-	-	-	-	-	-

* the values from references were read and calculated from the graphs and therefore do not indicate precise values.

Table S3. Tumor-to-heart ratios after administration of different FAPI conjugated radiopharmaceuticals in animal models at 1, 2, 4, 8, 17, 24, 48, 72, and 96h post injection.

	Precursor	Animal Model	Tumor/heart								
			1h	2h	4h	8h	17h	24h	48h	72h	96h
Dimeric	^{68}Ga-DOTAGA.(SA.FAPi)$_2$ [1]	PC3 CDX	0.9	1.2	-	-	-	-	-	-	-
	^{68}Ga-DOTAGA.(SA.FAPi)$_2$ [1]	U87MG CDX	0.8	1.0	-	-	-	-	-	-	-
	^{177}Lu-DOTAGA.(SA.FAPi)$_2$ [1]	PC3 CDX	-	-	2.0	-	-	2.1	2.8	2.2	2.6
	*^{68}Ga-DOTAGA.Glu.(FAPi)$_2$[2]	PC3 CDX	8.0	11.7	-	-	-	-	-	-	-
	*^{177}Lu-DOTAGA.Glu.(FAPi)$_2$[2]	PC3 CDX	-	-	16	-	-	14	28	35	-
	^{68}Ga-DO3A.Glu.(FAPI)$_2$[3]	PC3 CDX	19	24.5	-	-	-	-	-	-	-
	^{177}Lu-DO3A.Glu.(FAPI)$_2$[3]	PC3 CDX	-	-	12.5	-	-	18.8	31	35	50
	*^{177}Lu-BiOncoFAP-DOTAGA [4]	HT-1080.hFAP CDX	35	-	135	-	175	210	-	-	-
	^{177}Lu-BiOncoFAP-11 [5]	HT-1080.hFAP CDX	-	-	-	-	-	-	-	-	-
	^{68}Ga-DOTA-2P(FAPI)$_2$ [6]	HCC-PDX	4.0	-	-	-	-	-	-	-	-
	*^{177}Lu-DOTA-2P(FAPI)$_2$ [7]	HCC-PDX Model	1.6	-	2.1	-	-	4.0	3.8	-	-
	*^{177}Lu-DOTA-2P(FAPI)$_2$ [7]	HT-1080.hFAP CDX	11	-	12	-	-	-	-	-	-
	^{99m}Tc-TPPTS-HF$_2$ [8]	HT-1080.hFAP CDX	-	-	5.3	-	-	-	-	-	-
	^{99m}Tc-TPPTS-HF$_2$ [8]	U87MG CDX	4.8	6.3	5.5	-	-	-	-	-	-
	^{99m}Tc-L$_2$-TPPTS[9]	U87MG CDX	4.5	-	4.6	-	-	-	-	-	-
	^{99m}Tc-L$_2$-PDA[9]	U87MG CDX	7.2	-	5.3	-	-	-	-	-	-
	*^{68}Ga-LNC-1013 [10]	HT-1080.hFAP CDX	3.5	6.0	-	-	-	-	-	-	-
	^{177}Lu LNC-1013[11]	CT26-FAP CDX	-	-	1.9	-	-	4.7	6.3	8.3	3.6
	*^{68}Ga-FAPI-3 [10]	HT-1080.hFAP CDX	4.3	5.5	6.0	-	-	-	-	-	-
	*^{68}Ga-FAPI-5 [10]	HT-1080.hFAP CDX	6.8	4.0	-	-	-	-	-	-	-
	^{68}Ga-HBED-CC-FAPI-02 dimer [12]	U87MG CDX	14.2	10.2	-	-	-	-	-	-	-
	*^{18}F-ND-bisFAPI [13]	A549-FAP CDX	-	-	-	-	-	-	-	-	-
	^{177}Lu-ND-bisFAPI [13]	A549-FAP CDX	2.1	-	4.2	-	-	13.6	-	4.1	-
	^{177}Lu-FAPI-46-F1D [14]	HT-1080.hFAP CDX	-	-	18.7	-	-	12.8	-	13.2	-
	^{177}Lu-FAPI-46-F1D [14]	HEK-293.hFAP CDX	-	-	26.0	-	-	11.8	-	4.8	-
Trimeric	^{177}Lu-OncoFAP-23 [15]	SK-RC-52.hFAP CDX	-	-	-	-	-	240.7	323.0	344.2	492
Tetrameric	*^{177}Lu-TetraOncoFAP-DOTAGA [16]	SK-RC-52.hFAP CDX	-	-	-	-	-	-	-	-	-
	^{177}Lu-DOTA-4P(FAPI)$_4$ [17]	HT-1080.hFAP CDX	-	-	-	-	-	14.3	14.3	21.4	23.8
Hexametric	*^{177}Lu-HexaOncoFAP-DOTAGA [16]	SK-RC-52.hFAP CDX	-	-	-	-	-	-	-	-	-
Octameric	*^{177}Lu-OctaOncoFAP-DOTAGA [16]	SK-RC-52.hFAP CDX	-	-	-	-	-	-	-	-	-

* the values from references were read and calculated from the graphs and therefore do not indicate precise values.

References

1. Läppchen, T.; Bilinska, A.; Pilatis, E.; Menéndez, E.; Imlimthan, S.; Moon, E.S.; Afshar-Oromieh, A.; Rösch, F.; Rominger, A.; Gourni, E. Tailoring Fibroblast-Activation Protein Targeting for Theranostics: A Comparative Preclinical Evaluation of the 68Ga- and 177Lu-Labeled Monomeric and Dimeric Fibroblast-Activation Protein Inhibitors DOTA.SA.FAPi and DOTAGA.(SA.FAPi)2. *Molecules* **2024**, *29*, doi:10.3390/molecules29133093.

2. Martin, M.; Ballal, S.; Yadav, M.P.; Bal, C.; Van Rymenant, Y.; De Loose, J.; Verhulst, E.; De Meester, I.; Van Der Veken, P.; Roesch, F. Novel Generation of FAP Inhibitor-Based Homodimers for Improved Application in Radiotheranostics. *Cancers (Basel)* **2023**, *15*, doi:10.3390/cancers15061889.
3. Bilinska, A.; Kumar, N.; Gnesin, S.; Läppchen, T.; Menéndez, E.; Martin, M.; Rösch, F.; Rominger, A.; Gourni, E. DOTAGA.Glu.(FAPI)2 and DO3A.Glu.(FAPI)2 exhibit favorable preclinical thrannostic performance showing great promise for clinical translation: Preclinical evaluation and human dosimetry extrapolation for the lutetium-177 and terbium-166 counterparters. *in press* **2026**.
4. Galbiati, A.; Zana, A.; Bocci, M.; Millul, J.; Elsayed, A.; Mock, J.; Neri, D.; Cazzamalli, S. A novel dimeric FAP-targeting small molecule-radio conjugate with high and prolonged tumour uptake. *bioRxiv* **2022**, 2022.2002.2021.481260, doi:10.1101/2022.02.21.481260.
5. Puglioli, S.; Schmidt, E.; Pellegrino, C.; Prati, L.; Oehler, S.; De Luca, R.; Galbiati, A.; Comacchio, C.; Nadal, L.; Scheuermann, J.; et al. Selective tumor targeting enabled by picomolar fibroblast activation protein inhibitors isolated from a DNA-encoded affinity maturation library. *Chem* **2023**, *9*, 411-429, doi:10.1016/j.chempr.2022.10.006.
6. Zhao, L.; Niu, B.; Fang, J.; Pang, Y.; Li, S.; Xie, C.; Sun, L.; Zhang, X.; Guo, Z.; Lin, Q.; et al. Synthesis, preclinical evaluation, and a pilot clinical PET imaging study of (68)Ga-labeled FAPI dimer. *J Nucl Med* **2021**, doi:10.2967/jnumed.121.263016.
7. Zhao, L.; Chen, J.; Pang, Y.; Fang, J.; Fu, K.; Meng, L.; Zhang, X.; Guo, Z.; Wu, H.; Sun, L.; et al. Development of Fibroblast Activation Protein Inhibitor-Based Dimeric Radiotracers with Improved Tumor Retention and Antitumor Efficacy. *Mol Pharm* **2022**, *19*, 3640-3651, doi:10.1021/acs.molpharmaceut.2c00424.
8. Meng, L.; Fang, J.; Zhang, J.; Li, H.; Xia, D.; Zhuang, R.; Chen, H.; Huang, J.; Li, Y.; Zhang, X.; et al. Rational Design and Comparison of Novel (99m)Tc-Labeled FAPI Dimers for Visualization of Multiple Tumor Types. *J Med Chem* **2024**, doi:10.1021/acs.jmedchem.4c00772.
9. Ruan, Q.; Diao, L.; Li, Z.; Ding, D.; Han, P.; Jiang, Y.; Yin, G.; Feng, J.; Wang, Q.; Jiang, J.; et al. Design and preclinical evaluation of (99m)Tc-Labeled dimer FAPI-46 derivatives as potential tumor radiotracers. *Eur J Med Chem* **2025**, *287*, 117343, doi:10.1016/j.ejmech.2025.117343.
10. Tan, Y.; Li, J.; Zhao, T.; Zhou, M.; Liu, K.; Xiang, S.; Tang, Y.; Jakobsson, V.; Xu, P.; Chen, X.; et al. Clinical translation of a novel FAPI dimer [68Ga]Ga-LNC1013. *European Journal of Nuclear Medicine and Molecular Imaging* **2024**, doi:10.1007/s00259-024-06703-z.
11. Wang, P.; Zhang, H.; Zhao, L.; Zhang, Y.; Li, F.; Su, X.; Jing, H.; Zhao, L.; Zhang, J. Theranostic Potential of [(68)Ga]Ga/[(177)Lu]Lu-LNC1013: A Dual-Purpose Ligand for Cancer Imaging and Radionuclide Therapy. *ACS Appl Mater Interfaces* **2025**, doi:10.1021/acsami.5c07340.
12. Hong, H.; Zha, Z.; Zhao, R.; Luo, Y.; Jin, W.; Li, L.; Wang, R.; Yan, L.; Wang, H.; Ploessl, K.; et al. [68Ga]Ga-HBED-CC-FAPI Derivatives with Improved Radiolabeling and Specific Tumor Uptake. *Molecular Pharmaceutics* **2023**, *20*, 2159-2169, doi:10.1021/acs.molpharmaceut.2c01112.
13. Li, H.; Ye, S.; Li, L.; Zhong, J.; Yan, Q.; Zhong, Y.; Feng, P.; Hu, K. (18)F- or (177)Lu-labeled bivalent ligand of fibroblast activation protein with high tumor uptake and retention. *Eur J Nucl Med Mol Imaging* **2022**, *49*, 2705-2715, doi:10.1007/s00259-022-05757-1.
14. Millul, J.; Koepke, L.; Haridas, G.R.; Sparrer, K.M.J.; Mansi, R.; Fani, M. Head-to-head comparison of different classes of FAP radioligands designed to increase tumor residence time: monomer, dimer, albumin binders, and small molecules vs peptides. *European Journal of Nuclear Medicine and Molecular Imaging* **2023**, *50*, 3050-3061, doi:10.1007/s00259-023-06272-7.
15. Galbiati, A.; Bocci, M.; Ravazza, D.; Mock, J.; Gilardoni, E.; Neri, D.; Cazzamalli, S. Preclinical Evaluation of (177)Lu-OncoFAP-23, a Multivalent FAP-Targeted Radiopharmaceutical Therapeutic for Solid Tumors. *J Nucl Med* **2024**, doi:10.2967/jnumed.124.268200.
16. Galbiati, A.; Bocci, M.; Gervasoni, S.; Prodi, E.; Malloci, G.; Neri, D.; Cazzamalli, S. Molecular Evolution of Multivalent OncoFAP Derivatives with Enhanced Tumor Uptake and Prolonged Tumor Retention. *J Med Chem* **2024**, doi:10.1021/acs.jmedchem.4c01295.
17. Pang, Y.; Zhao, L.; Fang, J.; Chen, J.; Meng, L.; Sun, L.; Wu, H.; Guo, Z.; Lin, Q.; Chen, H. Development of FAPI Tetramers to Improve Tumor Uptake and Efficacy of FAPI Radioligand Therapy. *J Nucl Med* **2023**, doi:10.2967/jnumed.123.265599.
